# Photobiomodulation in Alzheimer’s Disease—A Complementary Method to State-of-the-Art Pharmaceutical Formulations and Nanomedicine?

**DOI:** 10.3390/pharmaceutics15030916

**Published:** 2023-03-11

**Authors:** Laura Marinela Ailioaie, Constantin Ailioaie, Gerhard Litscher

**Affiliations:** 1 Department of Medical Physics, Alexandru Ioan Cuza University, 11 Carol I Boulevard, 700506 Iasi, Romania; 2 President of ISLA (International Society for Medical Laser Applications), Research Unit of Biomedical Engineering in Anesthesia and Intensive Care Medicine, Research Unit for Complementary and Integrative Laser Medicine, Traditional Chinese Medicine (TCM) Research Center Graz, Department of Anesthesiology and Intensive Care Medicine, Medical University of Graz, Auenbruggerplatz 39, 8036 Graz, Austria

**Keywords:** bionanoformulations, brain, integrative nanomedicine, laser, light, nanomedicines, neurodegenerative disorders, photobiomodulation, cerebral photomedicine, picosecond laser stimulation, tight junction targeting

## Abstract

Alzheimer’s disease (AD), as a neurodegenerative disorder, usually develops slowly but gradually worsens. It accounts for approximately 70% of dementia cases worldwide, and is recognized by WHO as a public health priority. Being a multifactorial disease, the origins of AD are not satisfactorily understood. Despite huge medical expenditures and attempts to discover new pharmaceuticals or nanomedicines in recent years, there is no cure for AD and not many successful treatments are available. The current review supports introspection on the latest scientific results from the specialized literature regarding the molecular and cellular mechanisms of brain photobiomodulation, as a complementary method with implications in AD. State-of-the-art pharmaceutical formulations, development of new nanoscale materials, bionanoformulations in current applications and perspectives in AD are highlighted. Another goal of this review was to discover and to speed transition to completely new paradigms for the multi-target management of AD, to facilitate brain remodeling through new therapeutic models and high-tech medical applications with light or lasers in the integrative nanomedicine of the future. In conclusion, new insights from this interdisciplinary approach, including the latest results from photobiomodulation (PBM) applied in human clinical trials, combined with the latest nanoscale drug delivery systems to easily overcome protective brain barriers, could open new avenues to rejuvenate our central nervous system, the most fascinating and complex organ. Picosecond transcranial laser stimulation could be successfully used to cross the blood-brain barrier together with the latest nanotechnologies, nanomedicines and drug delivery systems in AD therapy. Original, smart and targeted multifunctional solutions and new nanodrugs may soon be developed to treat AD.

## 1. Introduction

Alzheimer’s disease (AD) is a pattern of progressive neurodegenerative disorder, described in 1906 by the German psychiatrist and neuropathologist Alois Alzheimer as “a special form of disease of the cerebral cortex”. The disease gradually sets in with the loss of the ability to remember recent events, slow thinking and expression and disorientation; then personality and behavior disorders appear, that become severe enough to significantly affect daily activities and the person’s ability to function independently [1,2].

The structural alterations of the brain in AD with increasing amounts of amyloid beta (Aβ) and phosphorylated tau proteins, simultaneously with the degradation of cerebral cortex neurons, facilitate the onset of dementia. Dementia is a generic term for a group of symptoms that refers to the deterioration of memory for recent events, language, problem solving, and the loss of other mental judgment skills. Dementia has many causes including: cerebrovascular disease, Lewy body disease, Parkinson’s disease (PD), hippocampal sclerosis, fronto-temporal lobar degeneration, “mixed pathologies”; however, AD is the most common cause (estimated at 60% to 80% of cases) of dementia.

In 2019, according to the World Health Organization (WHO) global health estimates, AD and various forms of dementia ranked seventh among the top 10 leading causes of death worldwide [3,4,5,6,7].

The WHO considers dementia a public health priority because it has important repercussions through special expenditures in health and social care. If at the beginning of 2020, the number of people with AD was almost 50 million people worldwide, and the global financial costs of people with dementia were USD 1.3 trillion, for the next period to 2030, it is expected that the number of people affected will continue to rise, and spending will exceed USD 2.8 trillion. Since the incidence of AD and dementia are directly proportional to age, the difficulties created by this disease are very high in most countries of the world; therefore, more valuable preventive and therapeutic measures are needed to reduce the burden of this the disease. In July 2021, WHO launched “Towards a dementia-inclusive society: WHO toolkit for dementia-friendly initiatives” and a multidisciplinary study plan for researchers and academics around the world to harmonize risk prevention methods, early diagnosis, treatment and innovative care [8,9,10].

Alzheimer’s disease is a big mental health problem, but also in terms of early diagnosis with the global aging of the population. Since 1984, the National Institute of Neurological and Communicative Disorders, Cerebrovascular Accidents and the Alzheimer’s Disease and Related Disorders Association have established the diagnostic criteria for AD; these criteria were updated in 2011 jointly by the National Institute on Aging and the Alzheimer’s Association. This useful recent guideline for the diagnosis of AD includes proposed elements for: clinically evident probable or possible AD dementia, and probable or possible AD dementia detected by pathophysiological parameters. Added to these diagnostic criteria is a category of biomarkers looking at brain amyloid studied by positron emission tomography (PET) and in cerebrospinal fluid (CSF) and markers of brain atrophy by nuclear magnetic resonance, neuronal damage, by investigating levels of phosphorylated tau (P-tau) and total tau (T-tau) in CSF and fluorodeoxyglucose for the metabolic activity [9,10,11,12,13,14,15].

The clinical onset of AD is the direct result of the reduction of neuronal mass in the cerebral cortex and the hippocampus area. The first signs that appear in a patient are: loss of recent memory, followed by confusion of previously known data and degradation of personality and mood, symptoms noticed in mild and moderate forms of AD. From a histopathological point of view, one could find extracellular amyloid deposits with the formation of plaques in the cortex, high concentrations of intracellular neurofibrillary tangles, reduction of neurons in the hippocampus, neuroinflammation, synaptic degeneration and impairment of cerebral lymphatic circulation; this clinically translates into memory disorders (e.g., the patient cannot remember what he did a few minutes ago) and/or concentration problems. MRI confirms cerebral atrophy, especially of the hippocampal area, associated with the increase in the amount of CSF and ventricular volumes [16,17,18,19].

Despite all the efforts undertaken by national and international organizations, the large number of studies and the increased incidence and prevalence, the etiology of AD is still unknown. Experimental and clinical studies indicate the multifactorial nature of the disease that includes: age (usually over 65 years), positive family history, genetics, hormonal disorders (the disease is more common in women), immune imbalances, mitochondrial dysfunction, cardiovascular problems, head injuries, diet (obesity, malnutrition, diabetes, etc.), smoking, alcohol abuse, sedentary lifestyle, sleep disorders, infections, and toxic environmental factors (heavy metals, trace metals, and others). Clinically, there are two types of AD: the early-onset type, which occurs in younger people, accounting for a small percentage of up to 5% of cases, and the late-onset type, known as loading or sporadic, which is the most common [20,21,22,23].

The role of genetic factors researched over the years is today unanimously accepted as having a major attribution in the onset of AD. In most cases, the disease with early onset in younger people is due to an autosomal dominant genetic factor, or to mutations of the genes that are involved in the processing of the amyloid precursor protein (APP), Presenilin-1 (PSEN-1), Presenilin- 2 (PSEN-2) and apolipoprotein E [9,24].

The first aim of this review was to reveal the dramatic situation and burden of AD for the medical system worldwide and to present aspects related to the pathophysiological mechanisms of this incurable neurodegenerative disease in order to address new advanced therapeutic strategies.

The second objective was to present and discuss state-of-the-art pharmaceutical formulations, development of new nanoscale materials, bionanoformulations in current applications and perspectives in AD.

The third goal was to focus on the latest scientific results in the literature on the molecular and cellular mechanisms of brain photobiomodulation (PBM) as a complementary method with implications in AD.

Another purpose of this review was to discover and to speed the transition to completely new paradigms for the multi-target management of AD, to facilitate brain remodeling through new therapeutic models and high-tech medical applications with light or lasers in the integrative nanomedicine of the future.

## 2. Pathophysiological Mechanisms in Alzheimer’s Disease

Summarizing the current publications regarding the diversity of pathogenic hypotheses in AD, one can mention the basic processes as: deposition of pathological proteins at the level of the cerebral cortex, neuronal loss with a decrease in neurotransmission, neuronal inflammation and oxidative stress. Despite all the progress made in unraveling the pathophysiology of AD, there is currently still no perfect understanding of the molecular mechanisms that lead to the accumulation of Aβ and tau proteins, and of the signaling pathways associated with neuronal and glial dysfunctions. Among the hypotheses of the pathophysiology mechanisms of AD, the ones that are unanimously accepted are: the dysfunction of microglia (protective cells of the nervous system) with the formation of amyloid plaques through the extracellular deposition of Aβ-peptides originating from APP, and the alteration of the structure of the fibrillar protein called tau, with the formation of aggregates of hyperphosphorylated proteins that disrupt the transport system and the functions of microtubules inside the neuron and the intracellular accumulation of a fine structure of neurofibrillary tangles (NFTs) [25,26,27,28,29].

The Aβ precursor protein is part of the cell membrane, being present in various tissues, acting as a surface receptor; at the brain level it has a special regulatory role in the formation of synapses, neuronal plasticity, antimicrobial function and iron export. As a result of the phenomenon of proteolysis of APP, Aβ is generated, a polypeptide that has in its structure between 37 and 49 amino acid residues, arranged in the form of amyloid fibrils, being the basic constituent of the amyloid plaques detected at brain examination of AD patients [30,31,32].

Studies in the last two decades support the hypothesis that in the pathophysiology of AD, the neurodegenerative alterations are directly related to the imbalance that occurs between the generation and elimination of Aβ1-42 peptides and, consequently, the neuronal storage of insoluble and toxic types of misfolded protein aggregates. Aβ peptide is commonly found in the central nervous system (CNS) as a degradation metabolite of APP, a unitary membrane protein involved in signal transduction in a complex pathway finely regulated by multiple enzymes and signaling molecules. Normally, the transmembrane peptide APP is disassembled by three enzymes: α, β, and γ-secretase. The largest proportion of APP is cleaved by α- and γ-secretases in the Aβ domain, releasing non-pathogenic non-amyloidogenic protein structures sAPPα and the C-terminal fragments (p3, carboxy-terminal fragment (CTF) 83 and AICD50) with 83 residues (C83), followed of C83 cleavage by γ-secretase.

In various pathological conditions or in the elderly, the reverse occurs, i.e., most APP follows an amyloidogenic cleavage pathway, with a first cleavage of APP by β-secretase, followed by cleavage by γ-secretase; then, the cleavage by β-secretase generates a soluble extracellular secreted domain, (sAPPβ), and the remaining membrane stub, C99, will undergo γ-secretase cleavage, releasing Aβ and its C-terminal counterpart AICD. After these biological processes, the proportion of Aβ peptide increases greatly and stimulates the formation of Aβ fibrils, which after accumulation, induce degeneration and death of the neuron. C99 dimerization, the conformation of C99 dimers and the cholesterol content of the neuronal membrane seem to play a key role in the development of sporadic AD. The data are supported by experimental studies that have shown that the accumulation of Aβ peptides in the brain can release reactive oxygen species (ROS), initiate the apoptosis cascade, and promote neurotoxicity [33,34,35,36,37,38,39,40].

Although the amyloid cascade hypothesis is accepted by most genetic, biochemical and clinical studies on the formation of amyloid plaques in AD, it failed to provide the expected results because the role of circulating Aβ oligomers (AβOs), which appear to be much more toxic, was not considered for the cognitive decline of the AD patient [41,42].

Today there is a broad consensus on the key role of AβOs in the generation of the neuronal destructive cascade, cognitive impairment and the extension of AD, but these AβOs show great variability in structure, size and cytotoxicity, so the study results are heterogeneous. Apart from the amyloid protein hypothesis, other studies demonstrate that tau protein, neuroinflammation and many other factors play a critical role in promoting AD [43,44,45].

Recent studies demonstrate that both toxic AβOs structures and toxic oligomers Tau (TauOs) can initiate prionoid-like neurodegenerative phenomena in AD; these toxic intracellular and extracellular oligomers and less amyloid plaques and NFTs are in the near future the target of new pharmacotherapies [46,47,48,49].

The human brain consumes a large part of the body’s energy and over 20% of the total oxygen metabolism; the basis of the disruption of this homeostasis is the mitochondrial dysfunction. Mitochondrial dysfunction and the imbalance between mitochondrial fission and fusion processes as well as bioenergetic deficits contribute to the death of neurons in AD patients; at the level of the mitochondrial membrane there are five protein enzyme complexes that participate in the release of ATP, namely: complex I, (NADH-ubiquinone oxidoreductase), complex II (succinate-ubiquinone oxidoreductase), complex III (cytochrome bc1 complex), complex IV (cytochrome c oxidase or CCO) and complex V (ATP synthase); all these structures have a low expression in the brain of patients with AD [50,51,52,53].

Within the blood–brain barrier (BBB), the presence of the two forms of monoamine oxygenase (MAO) A and B helps preserve the specificity of the function of catecholaminergic and serotonergic neurons and prevents the entry of potentially toxic “false neurotransmitters”. With aging, the balance between the two enzymes changes by increasing the concentration of MAO-B under the influence of genetic, environmental and hormonal factors and especially the proliferation of glial cells that accompany neuronal loss. MAO, as an enzyme imprisoned in the surface membrane of mitochondria, contains flavin-adenosine-dinucleotides (FAD) and participates in the degradation of biogenic amines into aldehydes and hydrogen peroxide with the consumption of molecular oxygen. Activation of this enzyme induces Aβ deposition through an abnormal cleavage of APP, causing neurofibrillary tangles and neuronal death with cognitive impairment. Following the catalytic action of MAO, hydrogen peroxide (H_2_O_2_) is released inside the reactive microglia in the brain tissue and the oxidative stress is unleashed with particular repercussions in neuronal degeneration [54,55,56].

Accumulation of Aβ in the brain is associated with oxidative stress, and Aβ in turn increases oxidative stress and promotes the onset of AD. During aging, the physiological antioxidant capacity decreases, so that a greater level of oxidized proteins, carbohydrates, lipid peroxides, toxic species (e.g., free carbonyls, ketones, peroxides), nuclear debris and mitochondrial DNA structures, leads to increase of ROS and at the same time of oxidative stress. Antioxidant therapy with vitamin C and E, even if it fails to provide effective neuroprotection, is still necessary in combination with other pharmacological or non-pharmacological methods. Another hypothesis in the pathogenesis of AD is neuroinflammation through the involvement of the partnership between microglia and astrocytes as a neuroprotective mechanism in the early stages of the disease through Aβ phagocytosis, then through the pro-inflammatory action of M1 phenotype microglia and A1 phenotype astrocytes, which release mediators with a cerebral neurotoxic effect [57,58,59,60].

The cholinergic hypothesis is discussed as the cause of AD pathophysiology because the gradual reduction of cholinergic neurons, sometimes over 75%, will have consequences on the secretion of acetylcholine (ACh), a CNS neurotransmitter with important roles in the transmission of impulses and the cognition process. ACh is the key chemical substance that mediates the transmission of the inter-neuronal signal, acts at the level of the neuro-muscular junction and mediates between the hormones produced by the endocrine glands. The main functions of Ach are to stimulate muscle activity, to regulate the cholinergic system, to assist cerebral neuroplasticity, to support higher cognitive functions (thinking, analysis, synthesis, attention), to regulate sleep and to protect the CNS from cognitive decline. ACh also modulates the release of other neurotransmitters such as dopamine, norepinephrine and serotonin. The number of receptors and transporters, as well as the secreted volume of norepinephrine and serotonin, are reduced in AD, resulting in reduced cognitive performance, memory loss, increased anxiety, agitation, aggression and other behavioral disturbances in dementia patients [61,62,63,64,65].

More and more evidence is emerging regarding the involvement of glucose metabolism and the disorders produced by the decrease in glutamate concentration with aging. There is evidence that the human brain contains approximately 86 billion neurons that are interconnected by electrical and chemical signals. An electrochemical stimulus, called an action potential, that occurs in the signaling neuron will release a series of molecular messengers into the synaptic space between two connected neurons. These messengers are then detected by the postsynaptic receptors of the receiving cell. *N*-methyl-d-aspartate receptors (NMDARs) and ion channels are located on the neuronal membrane both pre- and postsynaptically. Presynaptically located NMDARs are involved in the regulation of the release of the neurotransmitter glutamate. Glutamate, an ion derived from glutamic acid, is an important substance in cellular metabolism, the main excitatory neurotransmitter synthesized from glucose. Deviations from the physiological metabolism of glucose and glutamate in the brain are considered precursors to the deposition of Aβ plaques. More than 40% of neuronal synapses have been shown to be glutamatergic and the canonical hippocampal NMDARs control memory, the release of the transmitter glutamate and the brain-derived neurotrophic factor (BDNF). The *N*-methyl-d-aspartate receptor has a crucial role in synaptic transmission and plasticity, fundamental processes of the functioning of CNS and in learning and memory activity, but also in the phenomena of neurotoxicity. Increasing the amount of glutamate can cause overload of the post-synaptic response, thus facilitating the excessive penetration of Ca^2+^ into neurons and neurotoxicity. Overactivation of NMDARs and pathological Ca^2+^ signaling can lead to synaptic dysfunction and neuronal death with progressive decline in cognitive function and memory in AD patients. Therapies targeting the glutamatergic system represent a new hope for improving the living conditions of AD patients. A symptomatic and neuroprotective treatment in moderate to severe AD by memantine medication, a noncompetitive NMDAR antagonist that selectively blocks the function of extrasynaptic NMDARs, is U.S. Food and Drug Administration (FDA) approved [66,67,68,69,70].

## 3. State-of-the-Art Pharmaceutical Formulations and Nanomedicine Applied in Alzheimer’s Disease

For several decades, many treatment methods have been tested through clinical trials in AD, but until now the existing drugs act mainly on symptoms; these products have not yet truly proven their effectiveness in improving cognition, global behavioral function, quality of life and daily life activities. The discovery of new therapeutic formulas is vital for a better insight into the pathophysiological mechanisms and the implementation of a valuable treatment in AD. With all the complexity of the problems raised by patients with AD, there are currently only two established categories of pharmaceutical drugs approved by the FDA for the symptomatic therapy of this formidable pathology; these include cholinesterase inhibitors (naturally derived analogues, synthetics and hybrids) and NMDAR competitors. A third category approved very recently includes two monoclonal antibodies (aducanumab and lecanemab) directed against the buildup of Aβ plaques in AD brains [71,72,73,74].

### 3.1. Cholinesterase Inhibitors

Cholinesterase inhibitors (ChEIs) are chemical substances that prevent the degradation of acetylcholine or butyrylcholine, so they will lead to an increase in the amount of these products in the synaptic cleft and binding to muscarinic and nicotinic receptors. Acetylcholine (Ach) is the natural agonist of nicotinic and muscarinic receptors, being the mediator of the parasympathetic nervous system. In AD there is a continuous process of destruction of Ach-producing cells, with consequences in decreasing cholinergic transmission through the brain. The loss of cholinergic neurons from the cortex and hippocampus together with the reduction of cholinesterase activity are the main causes of degenerative changes in memory and cognition in patients with AD. Acetylcholinesterase inhibitor (AChEI) drugs (reversible, irreversible and pseudo-reversible) work by stopping the activity of the cholinesterase (ChE) and butyrylcholinesterase (BChE) enzymes. ChEIs are used as pharmaceutical drugs for myasthenia gravis and AD, but they are also used in agriculture as insecticides, and even as chemical weapons of mass destruction.

These pharmaceutical products are administered with the aim of improving cognitive functions and not to cure the patient. The drugs prescribed as ChE inhibitors include several products, such as: tacrine (TAC), donepezil (DP), rivastigmine (RSM) and galantamine (GAL). After more than two decades since the introduction of DP as the first cholinesterase inhibitor and then the other previously mentioned drugs approved in AD therapy, the results have not lived up to expectations. DP had several gastrointestinal (diarrhea, nausea, vomiting, decreased appetite), muscular (cramps, fatigue) and neurological side effects.

Neither GAL nor RSM, as AChEIs used in the symptomatic treatment of mild, moderate and severe AD, can modify or stop the progression of the disease, having the same side effects, less the formulations for transdermal administration [75,76,77,78].

Depending on the mechanism and duration of action, ChEIs are classified as short-acting or reversible drugs (e.g., TAC, DP and GAL), and intermediate-acting or pseudo-irreversible agents (e.g., RSM). In terms of the effectiveness of these ChEIs agents, it is similar, but the adverse effects, especially gastrointestinal, are important, which has led manufacturers to look for solutions to improve the pharmacological properties. Other research is directed toward their use in combination with other pharmacological agents, such as NMDARs antagonists, cholinergic precursors, and antioxidants [79,80].

Recent studies have revealed the extraordinary advantage of inventing and making different complex new pharmacological products formed from mixed molecules of natural origin. Since the etiology of AD is complex, current research is directed towards the development and establishment of therapeutic plans that include the use of pharmaceutical products that simultaneously hit several targets in the pathogenic chain. Remarkable benefits were obtained using “multi-target-directed ligands” (MTDLs), which reduced adverse effects, by improving the pharmacokinetic and pharmacodynamic profile of the drugs. Hybrid compounds made up of organic fragments, peptides, prodrugs and others, offer a greater disease compatibility through dual action, transport qualities, crossing the BBB and various milder actions on the cellular system [81,82,83,84].

Tacrine (Cognex^®^) was the first clinically approved ChEI, but due to the fact that it produced many side effects, it was abandoned in favor of other AChEIs. The qualities of the TAC drug have been enhanced by using the first hybrids from the propargylamine group (compound **1**), which increased AChEI activity and limited hepatotoxicity [85].

Chen et al. have prepared another hybrid compound based on silymarin (compound **2**), which has better neuro and hepatoprotective properties [86]. Tang et al. used oxoisoaporphine, which increased the anticholinergic activity and reduced the production of Aβ40-42 [87].

Other hybrids such as TAC-BF (compound **3**) further enhanced AChEI activity and significantly prevented Aβ aggregation; these have the ability to chelate metals (Cu^2+^ and Fe^2+^) and antioxidant properties [88,89].

Besides TAC, DP, RSM, many other conjugated or hybridized drugs for multi-target application in AD are being researched [90]. 

A synthesis of anticholinergic structure-based hybrids for the treatment of AD is shown in Table 1.

### 3.2. Antagonists of N-Methyl-d-Aspartate Receptors

Antagonists of NMDARs form the second large category of drugs approved by the FDA for patients with AD, but especially for those who present depressive, behavioral, psychotic disorders and different stages of dementia. At the level of the brain, glutamate is considered the primary neuronal excitatory transmitter, activating ionotropic and metabotropic glutamate receptors involved in synaptic transmission and neuronal plasticity, essential aspects in the memory and learning process. Excessive activation of glutamate receptors, in the case of NMDARs, will lead to synaptic dysfunction with an increase in Ca^2+^ permeability, which will work as a second messenger to modify synapses, and this excessive stimulation of glutamatergic signaling will lead to excitotoxicity. On the other hand, excitotoxicity produced by glutamate increases tau protein expression and phosphorylation. Activation of extrasynaptic NMDARs has also been shown to induce tau overexpression, degradation and reduced neuronal lifespan [66,110,111,112,113].

A valuable inhibitor of overactivated NMDAR is memantine, a drug that prevents disruption of axonal flow, neuronal necrosis, DNA fragmentation, nerve retraction, and tau phosphorylation. 

Memantine favors the activation of the NMDAR during the development of physiological memory and inhibits this receptor in the pathological situation of excitotoxicity. Memantine is proven to act as an antagonist for serotonin and nicotinic receptors. Memantine (Namenda, Namenda XR) has FDA approval for the ability to control AD symptoms in patients with moderate to severe AD, but also in those with mild to moderate symptoms who cannot tolerate acetylcholinesterase inhibitors. The clinical benefits of this NMDAR inhibitor have been proven to improve memory, functional communication skills, and daily routine activity. Administration of memantine in combination with AChEI is even more advantageous in patients with moderate to severe AD [114,115,116,117].

Namzaric^®^, a fixed-dose combination drug of donepezil and extended-release (ER) memantine, is currently approved by the FDA for patients with moderate to severe AD. This combination of cholinesterase inhibitors and NMDAR agonists interferes with the toxic effects produced by excess glutamate, together with those related to acetylcholine metabolism in the brain. The combination of memantine and donepezil was confirmed to be valuable compared to placebo or monotherapy in improving cognition, daily activities and neuropsychiatric symptoms in patients with moderate to severe AD symptoms [118,119,120].

With all the progress of the past decades in understanding the pathogenesis of AD, the increasing number of cases, the huge financial costs and the associated social problems, there is still no adequate treatment to delay the progression or reverse the disease. So far, the FDA has approved only the following seven drugs: donepezil, galantamine, rivastigmine, memantine, namzaric (a combination of memantine and donepezil), aducanumab, and most recently, lecanemab. The first five act symptomatically as cholinergic system agonists or NMDAR antagonists, and the last two, aducanumab and lecanemab as monoclonal antibody drugs in clearing Aβ plaques, that is, as Aβ-targeting antibodies to reduce their buildup [121,122,123].

### 3.3. Anti-Amyloid Monoclonal Antibodies Used in Alzheimer’s Disease

#### 3.3.1. Aducanumab

Aducanumab is the first biologic agent anti-amyloid monoclonal antibody rapidly approved by the FDA as a potential disease-modifying drug by targeting neuronal Aβ plaques, indicated in the treatment of AD. Aducanumab is a monoclonal antibody that acts on oligomers and fibrils (Aβ), reducing the amount of Aβ proteins, reducing the level of P-tau in CSF, as proven by PET, and thus it prevents cognitive decline [124,125,126,127,128].

There is opposition to the accelerated FDA approval of aducanumab (marketed as Aduhelm) for the treatment of AD, as safety and efficacy have not been guaranteed. Høilund-Carlsen et al. believe that the images from the amyloid-PET scans would actually represent an increased brain cell death and white matter destruction in the AD population due to aducanumab treatment, and by no means, a beneficial decrease in amyloid deposits [129,130].

Despite all the controversies regarding the biological agent aducanumab, there are some studies that demonstrate that it would have some clinical benefits by targeting the primary downstream anti-Aβ with direct upstream anti-tau repercussions, so that cognitive impairment is delayed [131,132,133,134,135,136,137].

#### 3.3.2. Lecanemab

After research and many testing studies undertaken by the Japanese pharmaceutical company Eisai, in partnership with the American company Biogen, the producer of the controversial Alzheimer’s drug Aduhelm, on 6 January 2023, the FDA granted accelerated approval of a new drug, Lecanemab, sold under the brand name Leqembi for the therapy of AD, a drug that would have the ability to slow down the cognitive decline of the patient when administered at the beginning of the disease. The route of administration is by intravenous infusion every two weeks, and the price of this new product would be lower than for Aduhelm. This treatment represents “an important advance in the fight to effectively treat Alzheimer’s disease”, which affects about 6.5 million Americans, the FDA said [138,139,140].

Lecanemab (trade name: Leqembi, other names: Lecanemab-irmb, BAN2401, mAb158) is a humanized form of the mouse IgG1 monoclonal antibody mAb158, which preferentially targets aggregated soluble Aβ, with activity on oligomers and protofibrils. Preclinical research showed that mAb158 significantly reduced Aβ protofibrils in the brain and CSF of Tg-ArcSwe mice [141], and further research in mouse neuron-glial cultures showed that mAb158 can reduce the toxicity of Aβ protofibrils by preventing pathological accumulation in astrocytes, because in human brain tissue it binds to diffusible Aβ fibrils [142].

Phase 3 research of the Clarity AD study with BAN2401 was reported on 27 September 2022, by Biogen and Eisai [143], as top positive results in all primary and secondary experiments, whereby two-thirds of the treated group became PET-amyloid negative at 18 months, and PET-tau revealed a significant reduction in tangle accumulation in the medial temporal lobe and trended downward in other brain regions. In the studied group there were three deaths due to hemorrhage; two patients had received blood thinners. Cerebral macrohemorrhages greater than 1 cm in diameter occurred in 0.6% of the treatment group and 0.1% of the placebo group [144].

Several other studies have shown that treatment with lecanemab led to a rapid and pronounced decrease in amyloid plaques, as well as a delay in clinical decline, as for example in a randomized double-blind trial of 854 subjects (lecanemab, 609; placebo, 245) with AD at onset, mild cognitive impairment, and mild AD dementia. Swanson et al. have used a Bayesian matched-response randomization design to evaluate 3 doses in 2 regimens of lecanemab versus placebo. After 12 months of treatment with the 90% effective dose (ED90) of 10 mg/kg biweekly, 64% of subjects were found to perform well, compared to 25% on placebo, on the Alzheimer’s Disease Composite Score. After 18 months of treatment with 10 mg/kg lecanemab biweekly, a decrease in brain amyloid accompanied by a consistent reduction in clinical decline was observed. Lecanemab was generally well tolerated with a 9.9% rate of amyloid-related imaging abnormalities—edema/effusion at the 10 mg/kg biweekly dose [145,146].

### 3.4. Side Effects of Drugs Approved for the Therapy of Alzheimer’s Disease

The use of acetylcholinesterase inhibitors (DP, RSM, GAL) and/or the non-competitive inhibitor of NMDAR (memantine) and anti-amyloid monoclonal antibodies (aducanumab and lecanemab) in dementia and AD, would be able to contribute to the temporary improvement or consolidation of the losses of memory and other cognitive functions, to the decline of behavioral disorders, and to limiting the patient’s dependence on aids; at the same time, however, these agents can bring great discomfort through the multitude of adverse reactions [147].

Acetylcholinesterase inhibitors, or the so-called anti-dementia drugs, through their action of excessive stimulation of cholinergic activity, peripheral muscarinic and nicotinic receptors, associated with the interference of other drugs, lead to a significant increase in the prevalence of adverse reactions including gastrointestinal (nausea, vomiting, diarrhea), neuropsychiatric (insomnia, restlessness, tremors, confusion, convulsions), allergic (cutaneous, respiratory), cardiovascular secondary to vagotonic effects (bradycardia, heart block, syncope), miosis, increased lens curvature, accommodation spasm, etc. [148,149,150].

Blockade of NMDAR with physiological disruption of glutamate in the brain can cause neurotoxicity of NMDARs agonists, and the appearance of dizziness, fatigue, hallucinations, drowsiness and confusion. Among all available AChEI drugs, donepezil therapy in combination with memantine appears to achieve more significant improvement in the general condition of the AD patient and with fewer adverse reactions [151,152,153,154].

The risk-benefit assessment of aducanumab reveals that the risks outweigh the clinical benefits, and the one-hour intravenous infusion, the very high price and the MRI imaging abnormalities (microhemorrhages and edema in the brain) related to cerebral amyloid raise a major safety issue for the patient. The fact that more money is needed for medical doctor’s evaluations and the cost of more MRI scans before and during treatment is another major impediment to widespread use of the drug. There are opinions that disagree with the FDA’s hasty approval of aducanumab because it raises major safety issues, lacks clinical evidence of effectiveness, is expensive and difficult to administer and monitor. The rejection of the marketing of aducanumab by the European Medicines Agency in December 2021 has attracted even more disputes about the unfair decision of the FDA [155,156].

Adverse effects during aducanumab administration are numerous and could be suggested by MRI images that can objectify the presence of amyloid-related imaging abnormalities of effusion (ARIA-E) and amyloid-related imaging abnormalities of hemorrhagic events (ARIA-H) expressed clinically by the state of mental alteration, confusion, delirium, disorientation, dizziness, headache, nausea, convulsions, vertigo, visual disturbances, with more frequent symptoms in apolipoprotein E4 carriers, advanced age, people with previous microhemorrhages, cardiovascular risk factors, etc. [127,157,158].

There are still concerns from the academic community about the safety of lecanemab because, similar to the warnings for aducanumab, there were also concerns for lecanemab about higher-risk cerebral edema and hemorrhages in patients with a genetic mutation, and the fact that during the phase 3 trial, some patients died. However, in a study published on 5 January 2023, van Dyck et al. demonstrated on 1795 enrolled participants with early AD, that lecanemab decreased amyloid markers at the onset of AD and triggered moderately less decline in cognition and function than placebo after 18 months of treatment but noted that there were side effects. Therefore, the authors mentioned that longer trials are still needed to demonstrate the efficacy and safety of lecanemab at the onset of AD [159,160].

A summary of the features of drugs approved by the FDA for the treatment of Alzheimer’s disease is presented in Table 2.

### 3.5. BBB and Types of Drug Carrier Systems in Alzheimer’s Disease

Protection of the CNS is provided by the BBB and CSF, ensuring its most favorable surroundings for proper functioning and homeostasis. There are selective biological barriers established by different cells at the following key interfaces: the BBB (cerebral vasculature), the blood-CSF barrier (choroid plexus), the brain-CSF barrier (pia arachnoid) and the CSF—brain barrier (neuroependyma). Without this very strong protection, the CNS would be exposed to a high risk of invasion by bacteria, viruses or other microorganisms, toxins, of ionic imbalance and other disorders, which would give rise to neuronal dysfunction and degeneration. The BBB provides both biochemical and immunological protection for the CNS. At this level there are different permeability thresholds for normal plasma components, toxic substances, bacterial agents and even difficulties in the penetration of some drugs. CSF, an ultrafiltrate of the choroid plexus from the cerebral ventricles, has roles of mechanical protection of the nervous tissue by absorbing shocks, of immunological protection and in the transport of nutrients, various molecules, hormones, drugs and harmful metabolic waste, which it removes. In the composition of the CSF there is a category of proteins with the role of influx and another category with the role of efflux of biomolecules. The BBB maintains a balance of the flow of biomolecules to and from the neuronal system of the brain and actively participates in the transport and regulation of Aβ peptides, their aggregation being also involved in the pathogenesis of AD. The direct connection between blood and brain structures is achieved through the chain of endothelial cells in the brain capillaries and the lymphatic system [189,190,191,192,193].

The BBB has a complex protective role of the brain and is at the same time a formidable barrier for even very small pharmaceuticals that cannot cross it, regarding which numerous nanotechnology-based research have been carried out in the last decade. The endothelial cells at the level of the BBB are tightly connected to each other by tight junction (TJ) proteins, very strong junctions which are formed by smaller subunits of transmembrane proteins (occludin, claudin, transmembrane adhesion molecules), which guarantee the selectivity of substances that can pass through this barrier, functioning as a diffusion sieve. This TJ multiprotein network directly controls the movement of solutes, ions, and water through the paracellular pathway [194,195,196,197,198].

In-depth studies on the ways in which various molecules or substances cross the BBB show that they can be organized into seven mechanisms, as follows: (1) passive diffusion across endothelial cells by a limited number of small molecules; (2) paracellular transport of limited water-soluble agents between endothelial cells, through TJ proteins; (3) active efflux transporters which mostly eliminate drugs and substances from the brain; (4) carrier-mediated transport which can be in either direction depending on the transporter and can be clatherin-dependent endocytosis. Major transporters include the glucose carrier, the L-type amino acid transporter **1** and **2**, cationic amino acid transporter **1** and **3**, the monocarboxylic acid carrier, the organic anion transporting polypeptide 1c1, the fatty acid transport protein **1** and **4**, the sodium-independent concentrative nucleoside transporter-2, the organic anion transporter **3**, organic anion transporter polypeptide 1a4 and 2b1, and the organic cation transporter **2.** (5) Receptor mediated transport relies on the interaction between ligands and receptors to transport larger molecules through the cells. These receptors include the transferrin receptor, insulin receptor, leptin receptor, lipoprotein receptor **1** and **2**, and the receptor for advanced glycation end products. (6) Absorptive-mediated transport is caveolin-mediated endocytosis and relies on the interaction between the ligand and the glycocalyx on the endothelial cells. (7) Ion transporters regulate the ions between the barrier and include sodium pumps, calcium transporters, and potassium channels [198].

Nanomedicine as an application of nanotechnology in medicine. It deals with the invention and production of nanomaterials with small dimensions similar to proteins and nucleic acids in the human body, which could interact with various biomolecules and cells to elucidate pathophysiological mechanisms, diagnose and enable targeted therapy. The European Commission created a definition for nanomaterial: it can be natural, incidental or manufactured and that can consist of aggregated or unbound particles, where one or more outer dimensions are between 1–100 nm for ≥50% of the particles [199]. 

Depending on the size, nanomaterials are classified into four different categories, as follows: zero-dimensional nanomaterials in which all three dimensions are on a nanometric scale (examples: quantum dots, fullerenes and nanoparticles, etc.); one-dimensional (examples: nanotubes, nanofibers, nanorods, etc.); two-dimensional (examples: nanowires, nanofilms, nanostructures, etc.); three-dimensional or bulk (e.g., bulk powders, dispersions of nanoparticles, networks of nanowires, nanotubes, etc. [200,201,202,203,204,205]. 

Systems for pharmaceuticals with the potential for easier BBB penetration and controlled release with multiple targets in the brain are already achieved by using various nanoparticles (NPs) based on lipids, polymers, inorganic components and other structures, used to prevent aggregation or sequestration of Aβ peptides in the brain of AD patients [206,207,208]. 

Drug delivery systems (DDSs) comprise a wide range of NPs, hydrogels, microformulations (microneedles, microparticles, microspheres and microemulsions) and NPs-loaded hydrogel (NLH) systems. Both NPs and microparticles can improve the therapeutic yield of drugs, protecting them from rapid degradation, improving their bioavailability and providing a more valuable release for the proposed site [187,209,210]. 

The concern for nanomaterials and especially for NPs has grown enormously in the last decades, due to the improved physical and chemical properties compared to the initial structure. These formidable characteristics have materialized in a wide range of innovative applications in the fields of medicine, pharmaceuticals, chemistry, agriculture, the food industry, electronics, etc.

NPs are small particles ranging from 1 to 100 nanometers in size, made of natural or synthetic polymers, lipids, metals, which are very small in size, have good loading capacity, encapsulation, efficient transport through the BBB and prolonged controlled release of the drug at the desired site. In the last decades, NPs synthesized “biologically” by using processes mediated by plants or microorganisms have appeared. The numerous advantages of DDS using NPs are confirmed by the FDA which approved the use of drugs combined with NPs with significant therapeutic potential in AD [204,211,212,213].

According to the structure, NPs can be classified into three classes: organic, carbon-based and inorganic. The category of organic NPs includes: proteins, carbohydrates, lipids, polymers or any other organic substances. The representatives of this category are liposomes, micelles, dendrimers and complex proteins such as ferritin; these NPs have non-covalent intermolecular structures, have stability, transport capacity, are sensitive to thermal and electromagnetic radiation, can be eliminated more easily from the body, and have no toxic potential; hence, they are preferred in biomedicine for the targeted administration of drugs [213,214].

Carbon-based NPs are composed of carbon atoms arranged in different shapes with dimensions below 10 nm, such as C60 fullerenes with the appearance of soccer balls. These NPs through the qualities of unique electrical conductivity, electronic affinity, optical, thermal and absorption properties, biocompatibility and reduced toxicity, serve for a wide range of applications: bioimaging, energy storage, photovoltaic devices and environment, pathogen detection, drug administration, etc. [215,216].

NPs manufactured from metal, ceramics or semiconductors have unique optical, electrical, thermal, magnetic and biological properties, that make them of great value in many chemical, biological, pharmaceutical, and biomedical applications [217,218,219,220].

Semiconductor NPs are made of semiconducting materials, which have properties between metals and non-metals. Ceramic ones are solids obtained from carbonates, carbides, phosphates, metal oxides and metalloids (titanium and calcium) and by their stability and high loading power are used in photonics, optoelectronics and biomedicine [221,222,223].

Metallic and semiconducting inorganic NPs, through their special physico-chemical properties, have an immeasurable capacity for use in the diagnosis and therapy of cancer, as well as in neurodegenerative diseases, due to the properties of light scattering and absorption through the localized surface plasmon resonance effect. Gold nanoparticles absorb light energy and transform it into heat, which it then releases into the environment. These optical properties can be used in the photodynamic therapy of cancer, of metastases, the improvement of tumor-specific magnetic resonance contrast, reversal of tumor-mediated immunosuppression, the targeted distribution of drugs, etc. [224,225,226].

Since there is a high failure rate in stopping or reversing the cognitive decline of AD patients by administering conventional therapy with the five drugs or biological agents approved by the FDA, new transport vectors and effective modes of action are being sought.

Nanoliposomes constitute a stable and promising drug delivery structure due to their biocompatibility and flexibility in transporting many different types of therapeutic molecules across the BBB and into brain cells. Assembling multiple structures together into multifunctional liposomes is another interesting area of research today [227].

The ability to destabilize the formation of Aβ fibrils in vitro (Aβ1-40 and Aβ1-42) and to reduce the expression of IL-1β, IL-6, GM-CSF and TNF-α was demonstrated using DP loaded on NPs of poly(d,l-lactide-*co*-glycolide) (50:50)-*b*-poly (ethylene glycol) [PLGA-*b*-PEG] [228].

Intravenously administered donepezil-loaded PLGA-b-PEG-NPs substantially inhibited acetylcholinesterase activity, ameliorating cognitive decline due to longer duration of action and strong destabilizing effect on amyloid fibrils [229].

Donepezil-loaded oil-in-water nanoemulsion (NE) is a new product formulated for intranasal administration. Studies by scintigraphy showed a maximum absorption of these NPs in the brain and could be an alternative for the use of donepezil hydrochloride in the treatment of AD [230].

RSM as a reversible, non-competitive inhibitor of AChE used orally has many limitations in effectiveness due to its poor stability and reduced ability to penetrate the BBB. Intranasal delivery of RSM by liposomes and cell-penetrating peptide modified liposomes improved the RSM distribution in brain and enhanced its pharmacodynamics and minimized side effects [171].

Nasal nanoliposomal RSM formulations present an improved systemic bioavailability, increased half-life and low clearance rate, compared to the classic oral formulation. RSM PLGA ameliorated scopolamine- and colchicine-induced memory deficits in animal models compared to oral RSM [172].

RSM mucoadhesive microspheres structured on chitosan, with polyvinyl alcohol surfactant solution, to which lecithin was added for nasal administration, confirmed strong bioadhesion on goat nasal mucosa and improved behavior and memory in Alzheimer rats compared to pure drug solution [173].

A promising therapeutic alternative to optimize the bioavailability of RSM in the management of AD are thermosensitive in situ nasal gels with nanostructured lipid carriers (NLC) and nanoemulsion loaded with an AChEI [174].

Chitosan thiolate NPs loaded with intranasally administered GAL were superior biochemically and pharmacokinetically, and inhibited AChE activity in the brain of Swiss albino mice compared to conventional oral administration of this drug in AD therapy [180].

Galantamine-bound chitosan nanoparticles (G-NPs) administered intranasally to a rat model of scopolamine-induced AD reduced Aβ deposition and suppressed Notch signaling, outperforming conventional GAL therapy [181].

The liposomal formulation for transdermal administration of RSM in the treatment of AD in experimental rats was a successful study as an alternative to the oral formulation, because the slow and continuous absorption reached optimal plasma levels in the therapeutic window [175].

Polymeric NPs are among the valuable competitors in drug delivery due to their nanosizing quality and inclusion of active molecules such as drugs, oligonucleotides and peptides, as well as due to the bioavailability, biocompatibility, bioactivity, controlled and sustained drug release, reticuloendothelial clearance, and lack of toxicity. Although these NPs are widely used, they also have some disadvantages, including low loading potential and drug capture efficiency. They could cause lysosomal ruptures through volume osmosis phenomena, which could induce mitochondrial dysfunction, aggregation, increase in oxidative stress and even complement activation, leading to an inflammatory process [231,232].

Memantine (MEM) loaded into biodegradable NPs below 200 nm with PLGA obtained by the double emulsion method and coated with polyethylene glycol MEM-PEG-PLGA decreased β-amyloid plaques and the associated inflammation characteristic of Alzheimer’s disease [233].

A class of lower generation poly(amidoamine) or PAMAM and lactoferrin conjugate loaded with MEM were investigated in vitro and in vivo in an aluminum chloride (AlCl_3_)-induced AD mouse model and revealed an improved bioavailability of MEM in the brain and a significant improvement in memory and behavioral responses [234].

Experimental study on neuropharmacokinetics and pharmacodynamics of RSM loaded on biodegradable NPs of methoxy poly(ethylene glycol)-*co*-poly(ε-caprolactone) (mPEG-PCL) administered intravenously in rats showed a linear integration plot; absorption clearance of the drug loaded in mPEG-PCL NPs was significantly higher than the free drug, and the animals recovered their memory losses much faster [176].

Hopes for the future are given by the research that is being done in the direction of the development of multifunctional liposome systems to target brain pathology, but also for the safe administration of drugs loaded on liposomes. Nanoliposomes have a composition similar to that of living cells, which gives them the advantage of posing fewer problems than other NPs made of polymers, silicon or metals, and they have the ability to easily cross the BBB and carry many types of molecules for blocking Aβ and/or tau aggregation. Multifunctional nanoliposomes are carrier systems with a great future for drug incorporation and delivery in the treatment of AD patients [227,235]. 

Other complex nanoparticle formulations have been used to treat Alzheimer’s disease in the mice models. An example is the messenger (m)RNA packaged polyplex micelle: expression of an anti-Aβ single-chain variable fragment (scFv) from mRNA-loaded polyplex micelle decreased the amyloid burden in an acute amyloidosis mouse model. This is a very interesting joint approach not only for AD therapy but also for immunotherapy against neurological disorders. The efficacy of the platform needed the intracranial delivery of anti-Aβ scFv as mRNA not DNA, as mRNA with an IL2ss secretion sequence to support the engagement of Aβ in the amyloidosis model, complexation with a smart copolymer for active transfection of primary neurons and to achieve detectable mRNA expression in the brain during 48 h. Amyloid burden decrease in this model was only achieved when all the three factors (mRNA coding scFv, smart copolymer, IL2ss) were incorporated into a single formulation [236].

Another example is the antibody fragments encapsulated polymeric micelle: a glucose ligand appended polymeric micelle loaded with 3D6 antibody fragments (3D6-Fab) successfully delivered a therapeutically efficacious level of 3D6-Fab into the brain parenchyma by crossing the blood-brain barrier for inhibiting Aβ1-42 aggregation in AD mice upon intravenous injection. This nanocarrier model is a hopeful procedure for efficient transportation of functional antibody vehicles to the brain and the therapy of neurodegenerative disorders [237].

Nanomedicine offers state-of-the-art alternatives to overcome the challenges of drug transfer across the BBB. A recent study exploited the impermeability of brain endothelium to selectively display the ligands on brain endothelial cells, followed by nanoparticle accumulation in the brain. Treatment of neurological abnormalities is severely hampered by the limited penetration of therapeutics/diagnostics into the brain due to the BBB, a highly impermeable cellular barrier composed of endothelial cell layer lining the cerebral micro- vessels. This high impermeability due to low endocytic rates of the BBB was exploited to retain ligands on the surface of the brain endothelium compared to peripheral endothelium. Nanocarriers capable of binding these displayed ligands are consequently directed specifically to the brain microvasculature. This selective brain targeting strategy decreased the “off-target” accumulation of nanocarriers to peripheral organs [238].

The future of nanomedicines for the management of AD requires the expansion of research, including on their clinical use. Nanotechnology and nanomedicine can be very valuable in efforts to counteract Alzheimer’s disease, but many in vivo studies are still needed for the safety of nanotherapeutic pharmacological compounds. At the same time, randomized multicenter studies on the pharmacokinetic and pharmacodynamic effects of drugs loaded on NPs and tested on humans are needed. Both gene therapy and immunotherapy are limited by many disadvantages, especially in restoring cognitive function and cognitive decline, but mostly through multiple side effects. Because of the failure of single-target drugs, combination therapies with multi-target potential seem to offer the best solution for the treatment of AD. Nanomaterials have already been shown to be capable of simultaneously delivering multiple drugs (e.g., chemical compounds, genes, peptides, and antibodies), thus offering the hope of a valuable option in AD therapy [239,240].

**Figure 1:**
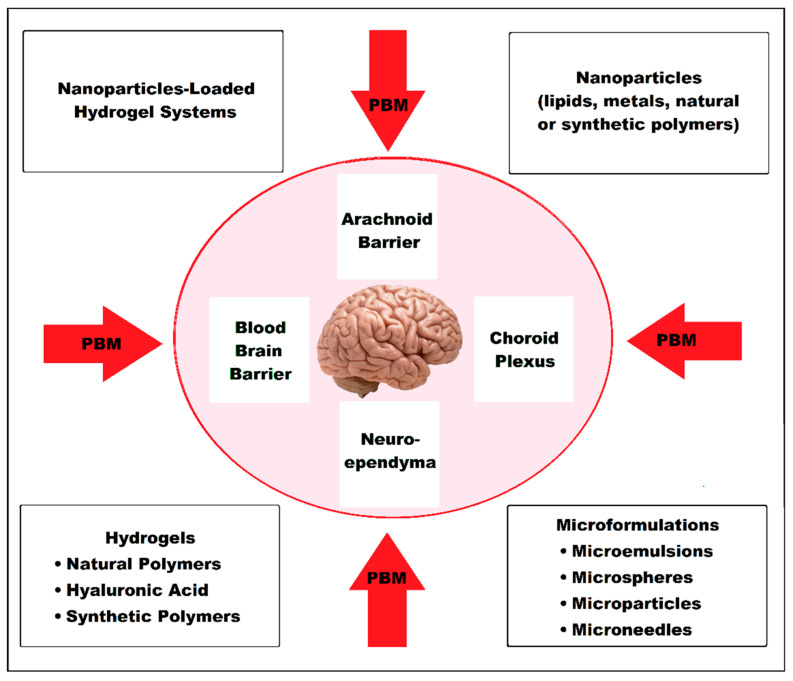
The possible management of AD and dementia to overcome protective brain barriers (arachnoid barrier, blood-brain barrier, choroid plexus and neuroependyma) with nanoscale drug delivery systems and PBM. (Figure 1 was imagined and drawn by L.M.A. using Microsoft Paint 3D for Windows 10 and using completely free picture material (Open—Human Brain Png) from SeekPNG.com (accessed on 31 January 2023), for which we are very grateful).

PBM and the main nanoscale drug delivery systems that could be applied in AD and dementia to overcome protective brain barriers are shown in Figure 1.

## 4. Light and Lasers in Medicine—A Brief Overview

Light as a healing modality goes hand in hand with human history and has probably developed over hundreds and hundreds of years, but contemporary PBM or photodynamic therapy (PDT) have only recently advanced following the discovery of the laser and its high-tech applications, such as the latest innovations in brain disorders, cancer or degenerative conditions, including AD [241,242].

Albert Einstein brilliantly predicted the phenomenon of stimulated emission as early as 1917, through a published work on the quantum theory of radiation, thus theoretically establishing the basis for MASER (Microwave Amplification by Stimulated Emission of Radiation) and LASER, acronym for Light Amplification by Stimulated Emission of Radiation, as recognized by the American Physical Society. A year earlier, Einstein turned his attention from general relativity to the interaction of matter and radiation, so he reimagined the fundamental statistical theory of heat, embracing the quantum of energy [243].

In 1964, the Nobel Prize in Physics was awarded to Dr. Townes, Dr. Basov and Dr. Prochorov, who “have made the atoms work for us in a new and most remarkable way. These magic devices called maser and laser have opened up vast new fields for research and applications which are being exploited with increasing intensity in many laboratories all over the world.” [244].

The discovery of the laser initiated a multi-billion-dollar industry, being applied in the arms industry, clinical medicine, different branches of surgery, cosmetics, sports, all industrial fields, optical communications and optical data storage, electronic devices and in almost all relevant fields of human activity, from supermarkets to entertainment. Since its first medical applications, PBM has been used for the treatment of many inflammatory diseases and especially for tissue regeneration and rehabilitation. The intensive development of advanced laser systems, light emitting diodes (LEDs), and other light devices has led to an unprecedented expansion of a multitude of therapy options, the value of which is the absence of or very few side effects, addressing precisely the energy processes inside the cells and what is more valuable—without toxic consequences [243,245].

More than half a century after the invention of the laser, PBM has become a complementary therapeutic method increasingly used in almost all fields of medicine and especially in medical recovery. Originally applied mainly for the treatment of superficial skin lesions and pain, dermatological conditions, cosmetic medicine or dentistry, today the use of PBM includes a plethora of pathologies, from diabetes, myocardial or cerebral infarction, brain trauma, spinal cord damage, peripheral nerve regeneration, neurodegenerative or chronic diseases, to modern applications with stem cells in regenerative medicine and the photoactivation of some pharmacological products with impact on the metabolism of living cells [246,247,248,249].

PBM, formerly known as “low-level laser (or light) therapy” (LLLT) remains today disputed as a therapeutic method due three main reasons. The first would be the insufficiency, ambiguity, confusion and lack of full understanding of all PBM mechanisms starting from the molecular, cellular and tissue levels; secondly, the design of a multitude of laser parameters that are very different in diverse PBM protocols (treatment time, wavelength, power density, pulse modulation, and so on); and thirdly, the biphasic dose response of PBM, that means that by increasing power density or treatment time, an inhibitory counter-effect could occur. This means that a higher laser dose does not necessarily mean better effects! Thus, the negative results in some studies can be explained, precisely because the parameters established in those studies were not well designed or chosen and proved ineffective [250,251,252].

Advent of much cheaper LEDs, which are not strictly monochromatic and lack the high coherence characteristic of lasers, has opened the way for new and multiple applications, but also ongoing disputes. Paradoxically, many of the applied PBM successes target our most complex organ, the brain, and relate to the CNS where many particularly serious conditions or injuries can be treated non-invasively by transcranial PBM or tPBM; successes are also seen in the peripheral nervous system for nerve regeneration and pain relief [251].

In the following, we will focus on cellular and molecular mechanisms in neurodegenerative diseases, with a special focus on AD.

## 5. Photobiomodulation of the Brain and the Treatment of Alzheimer’s Disease

Today’s medicine must deal with alarming health problems, among which, after cancer, the category of neurodegenerative diseases, including AD, puts increasing pressure on the world health system. Degenerative brain diseases can evolve at a slow pace, but so far contemporary medicine has not been able to successfully treat them or stop their irreversible progression. In general, degeneration of the brain, as a particularly complex organ, requires an interdisciplinary approach so that PBM could be a complementary non-invasive solution.

For example, the neuroprotective effect of a new photobiomodulation technique against Aβ25-35 peptide-induced toxicity in mice recently suggested a new hypothesis for the therapeutic approach of AD [253].

Photobiology has many objectives, an essential one being deciphering and highlighting the effects of polychromatic light on living systems. Unlike modeling the dose-response as a linear function of incident light at each wavelength in the spectrum, the action resulting from a multi-wavelength spectrum is much more complicated and should be calculated by integrating the cross-sectional product for the response at each wavelength and spectral irradiance at that wavelength, both over wavelength and time, results that are true only if the dose-response functions are linear with respect to photon dose. There are multiple linear photochemical reactions in relevant dose ranges, but many final biological reactions, especially in the case of cell survival, no longer obey linear laws. In the living world, many coupled light-driven reactions are governed by intricate non-linear dose-response rules, from which we are currently increasingly trying to design dosimetry guidelines, including for AD [254,255].

PBM consists of applying electromagnetic radiation with wavelengths in the visible range (400–700 nm) and in the near-infrared (NIR) (700–1100 nm) range with a low power between 1 mW and 1 W, so without thermal or mechanical effects, as a complementary method to restore health, working towards the intended goal to biostimulate the targeted cells and tissues. The result is the initiation of some photochemical reactions in the cells, the method being called biostimulation or PBM, in order to relieve pain and inflammation, prevent cell and tissue apoptosis, support recovery from illness, and ultimately, if possible, cure different diseases or disorders. To produce a result on living mammalian cells, photons from the PBM process must be absorbed by the electronic absorption bands belonging to a molecular chromophore or photo acceptor. A chromophore is that fraction of a molecule responsible for its color, where the energy difference between two separate molecular orbitals falls within the range of the visible spectrum. When light hits the chromophore, it can certainly be absorbed by exciting an electron from its ground state to an excited state, so the chromophore will cause a conformational change in that molecule [256,257].

An example of absorption of light of different wavelengths inside living cells is a phenomenon with effects in the mitochondrial respiratory chain. It has been proven that the first complex, or complex **1** (NADH dehydrogenase) absorbs blue and ultraviolet light, the third complex or complex **3** (cytochrome c reductase) absorbs green and yellow light, and the fourth, i.e., complex **4** (cytochrome c oxidase or CCO) absorbs red and infrared light [258,259].

In the first complex or NADH dehydrogenase, the flavin prosthetic set and the iron sulfur clusters have operational prominence. The flavoproteins contain the nucleic acid derivative riboflavin found in the flavin adenine dinucleotide (FAD) and the flavin mononucleotide (FMN). Riboflavin is characterized by high fluorescence which allows light absorption and is vital to the configuration of these two major coenzymes, FMN and FAD, involved in many living modi operandi such as cellular energetic metabolism, cellular respiration, antibody production, etc. [260].

The vast instability of the redox and catalytic features of the enzymes in different organisms may be due to adjustment to the specific surroundings in which these enzymes are working [261].

In redox reactions, the flavin coenzymes assist the action of more 80 flavoenzymes in the human organism (and even hundreds more across other species, including those enciphered by an archaeal, bacterial, fungal complete haploid set of chromosomes and its associated genes). Two thirds of human flavoproteins are linked to human diseases and the flavoenzymes are essential for the biosynthesis of other coenzymes and hormones [262].

For example, results from animal and cellular models suggest that FAD-deficient forms of NAD(P)H quinone oxidoreductase **1** (NQO1) may accelerate the aggregation of Alzheimer’s Aβ peptide (Aβ1-42). Aβ1-42 alone forms rod-shaped fibril structures, whereas in the presence of NQO1 isoforms, Aβ1-42 is incorporated in the middle of larger globular protein aggregates surrounded by NQO1 molecules, indicating the potential relevance of FAD-deficient forms of NQO1 in the amyloid aggregation diseases [263].

Mitochondria are the energy generators of the eukaryotic cell, converting oxygen and nutrients through oxidative phosphorylation and the electron transport chain (ETC) into adenosine triphosphate (ATP). High-energy electrons pass through a series of transmembrane complexes, including CCO to the final electron acceptor, generating a proton gradient, the latter being used to produce ATP. Multiple in vitro experiments demonstrated an upward regulation of cellular respiration when mitochondria were exposed to various forms of lighting, simultaneously with the increase of products such as ATP, NADH, ribonucleic acid (RNA), proteins and others, but also the increase of oxygen consumption [251].

For example, one research group applied to two groups of 10 healthy participants 8 min of PBM with different lasers at 800 nm, 850 nm, and 1064 nm, as well as an LED at 810 nm on the human forearm in vivo to measure the effects on vascular hemodynamics and CCO redox activity, measured before, during, and after active or sham PBM. Pruitt et al. investigated whether different laser wavelengths could determine distinct PBM effects, and if a LED at a similar wavelength to a laser could induce similar PBM effects. A broadband NIR spectroscopy system was used to assess concentration changes in oxygenated hemoglobin Δ[HbO] and oxidized CCO, Δ[oxCCO]. The results proved that all three laser wavelengths triggered significant increases in both Δ[HbO] and Δ[oxCCO], while the 1064 nm laser supported the increases longer, and that the 810-nm LED with a medium irradiance of 135 mW/cm^2^ induced measurable and significant increases in both parameters, compared to placebo [264].

The impact of light on living cells was proposed by Karu and begins with the absorption of visible or near-infrared photons by CCO chromophores, followed by a change in the mitochondrial membrane potential, an increase in ATP synthesis and increased levels of ROS, NO and calcium ions, phenomena that could remodel the interaction between mitochondria and the nucleus. This will alter the ultrastructure of mitochondria with direct repercussions on the intrinsic dynamics of mitochondrial fission and fusion processes, changes in ADP production, redox potential, pH, intracellular cAMP values, which will influence the activator protein-1 and NF-kB, membrane permeability and ion fluxes. Interdependent pathways have been proposed regarding the direct regulation of some genes, all these aspects being included under the umbrella of retrograde mitochondrial signaling [265].

Analyzing the action spectra in the range 600–1100 nm for different biological cells, Karu et al. demonstrated that monochromatic radiation is able to trigger photobiological processes in the cells and that there are four “active” regions in all spectra, even if the maximum is not always the same; it was assumed that the fundamental molecular mechanisms would be the modulation of mitochondrial CCO activity [265,266,267].

In fact, the mitochondrial signaling is an information channel between the mitochondrial respiratory chain and the nucleus to transduce signals regarding the functional state of the mitochondria, where CCO, as the final enzyme in this respiratory chain, functions as both a signal generator and a signal transducer [268].

As the final enzyme of the ETC, CCO has been shown to be a photoacceptor and photosignal transducer in the red (R) and NIR regions of the electromagnetic spectrum and allows the relocation of the electrons from cytochrome c to molecular oxygen, thereby increasing MMP, ROS, cyclic adenosine monophosphate (cAMP) and ATP levels [269].

ROS rapidly modulate gene expression by activating different transcription factors and signaling pathways, for example NF-κB, a redox-sensor, which has key functions for neurons in signal transfer, adjustment of intrinsic cyclic processes, production of active enzymes, nucleic acids and new protein synthesis, etc. Oxygen metabolism triggered by PBM will increase ATP which results in a brief discharge of ROS, which in reverse activates transcription factors, upregulating different stimulating and protective genes for cell proliferation and survival, as well as cell migration, growth factors, etc. When PBM is completed, the cascade stimulated by transcription factors will not end right away. ROS action is a Janus-faced mediator, helpful in reduced concentrations or brief exposures, and noxious at high concentrations or with long-term action [257,270]. Oxygen is the last acceptor in the ETC through which 90% of oxygen absorbed by cells is consumed to produce ATP, while only an insignificant quantity of oxygen is converted into ROS. Many experimental AD studies and a few clinical trials have highlighted the significant potential of PBM for AD therapy. R and NIR light at a low dose can successfully decrease the accumulation of Aβ-plaques in the CNS. Connection dose–response directly controls the therapeutic effect in AD. Discovering the best PBM parameters for AD to eliminate Aβ plaques and ameliorate AD clinical signs is a clear challenge to improve its efficacy [253,270,271].

Karu discussed the multiple roles of CCO in mammalian cells under R and NIR irradiation and highlighted the critical role of ATP as a signaling molecule [272].

In fact, in humans, 4 electrons are remoted from 4 molecules of CCO and ceded to O_2_ along with 4 protons, thus resulting in 2 molecules of H_2_O. Simultaneously, 8 protons are relocated from the mitochondrial matrix, of which only half are translocated across the membrane, increasing the proton gradient. The whole picture of the proton pump in CCO is as yet under investigation. Peter D. Mitchell won the Nobel Prize in Chemistry in 1978 for his discovery of the chemiosmotic mechanism of ATP synthesis, i.e., the coupling by a proton gradient across the inner mitochondrial membrane between the ETC and the oxidative phosphorylation [273].

Stopping ATP synthase leads to a gradual increase in protons and, consequently, a higher proton-motive force, thus causing the reverse flow of electrons [274].

PBM enhances the operation of all complexes I, II, III, IV and V of the ETC. CCO (or complex IV) is the primary photoacceptor because the main oxygen consumption occurs at this site during the action of PBM. In addition to increasing ATP and cAMP, the level of nitric oxide (NO) will be increased by release from metal complexes in CCO; photodissociation of NO from its binding sites in CCO reverses mitochondrial inhibition of cellular respiration and facilitates ROS generation. NO suppresses mitochondrial respiration through various well-investigated processes and several nitrogen derivatives. It has been shown in cultured cells and tissues that low NO precisely and reversibly instantly inhibits CCO in competition with oxygen; meanwhile, higher NO and its derivatives can induce irreversible suppression of RCT and/or cell death. Hence, the inhibition of CCO by NO may be involved in the physiological and/or pathological adjustment of respiration rate and its affinity for oxygen, which depends on different factors such as pH, proton motive force, supply of O_2_ to cells and tissues, as well as the generation of reactive nitrogen species [275].

It has been proposed that both structural and functional changes of the crucial interaction in the R and NIR domains with mitochondria could lead to the complex effects of PBM. However, additional to CCO, many other biomolecules inside mitochondria or other parts of cells (proteins, nucleic acids, and so on) are sensitive to light and could undergo important changes in their biochemistry. Comprehensive elucidation of the processes of light interaction with biological systems is yet to be completed for extensive application in photomedicine or industrial applications [276].

Another model would be related to photons interacting with water interfacial layers and participating in ATP regulation. The interplay between ATP upregulation and ROS downregulation in oxidatively stressed cells could be interpreted in a new way, according to a recently published paper. Based on the abundant evidence that different R-NIR wavelengths, delivered by lasers or LEDs, are effective in up-regulating mitochondrial ATP levels through the interaction of photons with mitochondrial bound water and interfacial water layers, the author of a recent study suggests the interplay between light-induced changes in the following important physical parameters in biostimulated cells: density (expansion in volume), viscosity (decrease) and the interfacial tension (presumed reduction, but harder to interpret as it is a function of the first two parameters) between bound water and the surrounding bulk water matrix [277].

Regarding the mechanisms of PBM, researchers are looking for new answers and proposing new models [278].

A synthesis of molecular and cellular mechanisms resulting from PBM action on living cells, in neurodegenerative diseases, as well as recent advances and challenges in AD have been presented in various reviews [241,257,259,279,280].

New insights into light-matter interactions are being advanced by interdisciplinary teams, modeling light-induced forces in systems with tens of thousands of atoms and arbitrary nanostructures. Ambrosetti et al. demonstrated the existence of both types of mechanical forces (attraction and repulsion) in the interaction of light with molecules, i.e., both attractive and repulsive optical van der Waals (vdW) forces can be induced by light, so generating new frameworks for the activation and the command of the living dynamic processes by light. Surprisingly, extensive analysis of the human formaldehyde dehydrogenase protein revealed both types of mechanical deformations (localized and delocalized) that depend mainly on photon energy. So, at low energies, photons activate only local deformations, while at higher energy, photons are able to initiate large-scale motions of this protein, i.e., the absorbed light could trigger productive energy transfer and selectively activate collective molecular vibrations.

The deep understanding of light-matter interactions in living complex molecular systems is one of the ultimate challenges of science today, especially for medicine, physics, chemistry, biotechnologies and so on. In fact, Ambrosetti et al. have opened the door for novel practical and precise treatments with photon-induced mechanical vibrations in large molecules, with a huge potential to investigate a multitude of new, previously inaccessible phenomena [281].

In current research for the management of brain diseases, noninvasive procedures (which do not require the introduction of instruments into the brain, i.e., do not invade the brain) that are simply directed to a specific area, without effort, without unwanted repercussions, are increasingly used for fast tracking, in decoding the relationship between brain regions, their particular activities and connections to patient behavior and final results. One of these noninvasive brain stimulations is PBM. With a non-heating action, the photons from PBM engage chromophores in tissues or cells and trigger physical and chemical photo effects at multiple living levels. Regardless of the extensive proofs for benefits in the complementary management of many medical conditions, a comprehensive insight into the fundamental, intrinsic healing processes is not yet available, especially in brain disorders such as Alzheimer’s disease, depression and so on.

Anti-beta-amyloid and anti-tau antibody therapy, vaccines, and other methods of reducing tau and/or amyloid have not lived up to clinical expectations after all the efforts of researchers in the pharmaceutical and nanobiotech fields. 

An achievement with major prospects from the scientific community seems to be the idea of using laser devices or LEDs that emit radiation in R-NIR range. PBM can activate molecules and modulate intracellular pathways, pumps, and biochemical reactions, with favorable short- and long-term consequences, both local and systemic. Transcranial or intranasal PBM for brain stimulation with R and NIR light has focused attention on positive outcomes on cognitive functions, the repair of neurodegenerative processes, control of energy metabolism, and adjustment of chronic brain inflammatory processes in AD subjects. Publications of experimental and clinical studies on tPBM in AD and other neurodegenerative disorders and ischemic brain injuries have demonstrated that this modality of therapy stimulates metabolic processes in brain tissue, increases intracellular ATP production and revives neuronal mitochondrial activity; modulates local oxidative stress; favors angiogenesis, capillary and lymphatic revascularization; restores destroyed synapses and generates new synapses; revitalizes the growth of new neural cells and acts as a neuroprotector, thus improving cognitive function and memory [282,283,284,285,286,287,288,289,290].

A summary of current studies on PBM applied to the CNS in human subjects is presented in Table 3.

The outstanding effects of PBM on the CNS, regardless of the administration method, can be considered as a “green” drug in Alzheimer’s disease and dementia. The monitoring of patients for long periods of time, up to 15 years, has objectively shown positive results in all cases. For example, after many years from the transcatheter intracerebral stimulation, conclusive images proved neurogenesis inside the cerebral areas with the complete restoration of the lobes and normal blood supply to the brain due to angiogenesis, aspects clearly highlighted by CT, MRI and Magnetic Resonance Angiography (MRA); from a clinical point of view, there was rapid recovery of memory, reduction of the severity of dementia and an improvement of cognitive functions throughout the monitoring period [310].

**Figure 2:**
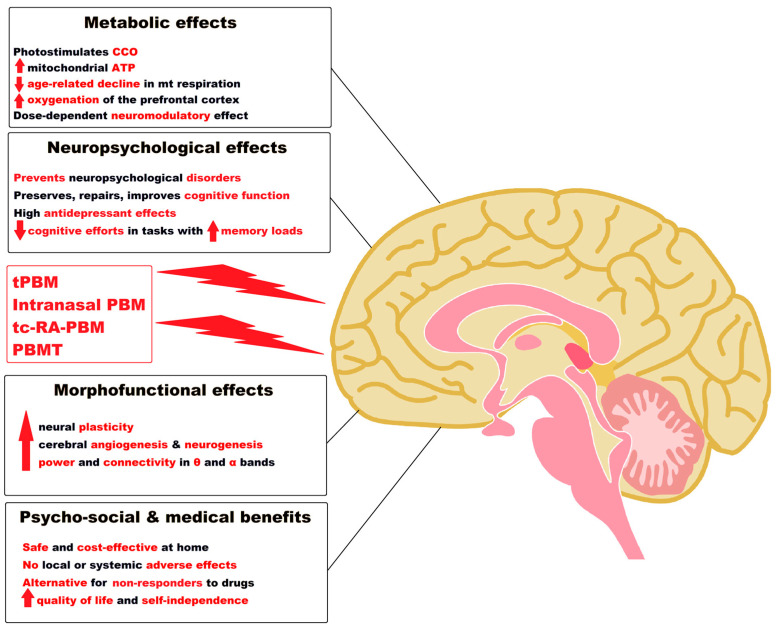
Notable effects of tPBM, intranasal PBM, tc-RA-PBM and PBMT as a “green” medicine in Alzheimer’s disease and dementia. Legend: ATP = Adenosine triphosphate; CCO = Cytochrome c oxidase; PBM= Photobiomodulation; mt = mitochondrial; tPBM = Transcranial PBM; tc-RA-PBM = Transcutaneous radial artery PBM; PBMT= Transcatheter Intracerebral PBM; α = alpha; θ = theta; ↑ = increase; ↓ = decrease. (Figure 2 was imagined and drawn by L.M.A. using Microsoft Paint 3D for Windows 10 and using completely free picture material (Open—Human Brain Png) from SeekPNG.com (accessed on 31 January 2023), for which we are very grateful).

An illustrative diagram of the remarkable effects of PBM applied in various ways and with different technologies in AD, as evidenced by results from the recent human studies summarized in Table 3, is presented in Figure 2.

Recent advances in tPBM and cutting-edge breakthroughs in AD treatment are opening previously unimagined perspectives. 

Reversible modulation of the BBB by transcranial picosecond laser stimulation with molecular-targeted gold nanoparticles (AuNPs) has recently been demonstrated experimentally. Li et al. advanced a simple nanotechnology by applying picosecond-laser excitation of TJ-targeted AuNPs for BBB modulation. The researchers experimentally tested the possibility of targeting a TJ complex in vivo. They selected 50 nm spherical AuNPs with a surface plasmon resonance peak around 530 nm, which perfectly matched with the 532 nm picosecond laser used. As a result, the paracellular permeability of the BBB was reversible and gradually increased, thus allowing the systemic delivery of immunoglobulins and other particles to the brain without affecting the dynamics of blood circulation or damage to neurons, thereby opening innovative avenues for noninvasive therapeutic interventions in the CNS [312]. 

This latest experiment demonstrates how tPBM could be successfully used to cross the BBB together with the latest nanotechnologies, nanodrugs and DDSs in AD therapy.

A better understanding of the interaction and effects of PBM on the targeting of TJs and the molecular mechanisms involved in the modulation of BBB permeability can have a huge impact on future understanding, ranging from the pathophysiology of neurodegeneration to autoimmunity [313].

## 6. Concluding Remarks and Future Perspectives

Alzheimer’s disease is the most common neurodegenerative disorder and today represents a major challenge for medicine due to the aging of the population, the latest lifestyle trends, the large number of affected people, and the impact on the daily life of patients and their caregivers, but especially due to the financial repercussions it produces. Worldwide, there are more than 50 million people with neurodegenerative conditions that burden society, as every 3 s the number increases with another case, and thus the number is expected to triple by 2050, if preventive measures are not taken and new effective therapeutic means are not found.

As a multifactorial disease, the origins of AD are not satisfactorily understood, and despite huge medical expenditures and attempts to discover new pharmaceutics or nanomedicines, there is no cure for AD, and not many successful treatments are available to delay the progression or to reverse the disease. So far, the FDA has approved only seven drugs: donepezil, galantamine, rivastigmine, memantine, namzaric (a combination of memantine and donepezil), aducanumab, and most recently, lecanemab—the last two being monoclonal antibodies.

Transcranial PBM has recently emerged as a potential clinical treatment and cognitive enhancement method for various neurodegenerative pathologies by delivering red and near-infrared light to the frontal and midbrain areas, which could also help increasing the potential of pharmacological therapies.

New insights from an interdisciplinary approach, including the latest results from PBM applied in human clinical trials such as tPBM, tc-RA-PBM or PBMT (intracerebral transcatheter), combined with the latest nanoscale drug delivery systems to easily overcome the brain’s protective barriers, could open new perspectives for rejuvenating our CNS, the most fascinating and complex organ.

As nanomedicine has evolved dramatically over time and the field continues to advance at incredible speed today, state-of-the-art drug delivery systems and approaches are growing and advancing instantaneously.

A brilliant partnership between the laser/light industry and the future of clinical drug development in the light of nanotechnologies offers hope to patients with AD and dementia.

tPBM with near-infrared (NIR) light may be a promising non-pharmacological treatment for cognitive impairment in AD. Due to its low cost, safety profile, and ease of self-administration right at home, tPBM has the potential to become widely accessible.

**Figure 3:**
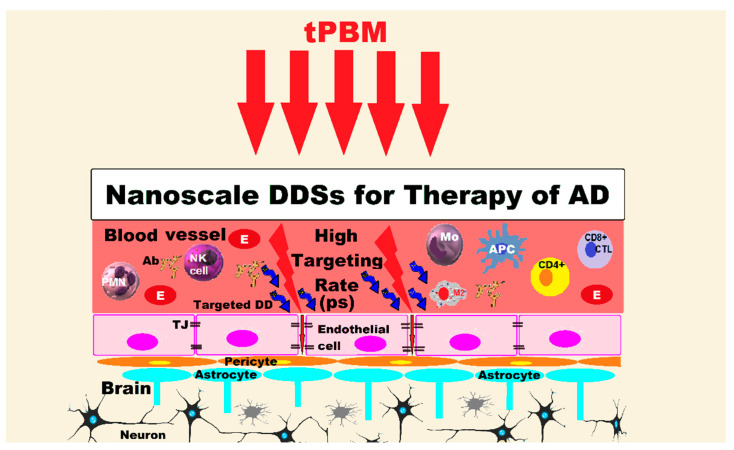
Breaking the BBB: future innovations with picosecond laser excitation and nanoscale DDSs in AD. Legend: Ab = Antibody; APC = Antigen presenting cell; DD = Drug delivery; DDSs = Drug delivery systems; CD4+ = CD4+ T lymphocytes; CD8+ = CD8+ T lymphocytes; E = Erythrocyte; ps = Picosecond; PMN = Polymorphonuclear neutrophil; M2 = Activated or healing macrophage of the M2 type; Mo = Monocyte; NK cell = Natural Killer cell; TJ = Tight junction; tPBM = Transcranial PBM. ↓↓ = Direction of paracellular transport. [Figure 3 was imagined and drawn by L.M.A. using Microsoft Paint 3D for Windows 10 and using completely free picture material (Open—Human Brain Png) from SeekPNG.com (accessed on 27 February 2023), for which we are very grateful].

Recent advances in tPBM and cutting-edge breakthroughs in TJ-targeting of BBB endothelial cells, together with state-of-the-art DDSs open previously unimagined perspectives in the treatment of AD, as we imagined and illustrated in Figure 3.

For innovative solutions, researchers need to rethink time in picoseconds and rely on the latest applications of laser stimulation and state-of-the-art nanoscale DDSs so that in the near future we can successfully treat AD. 

Only by adding the fourth axis, time in picoseconds, along with thinking of matter at the nanoscale, will we be able to revolutionize medicine.

Original, smart and targeted multifunctional solutions as well as novel nanomedicines could soon be developed to treat AD and dementia.

## Figures and Tables

**Table 1:** Anticholinergic structure-based hybrids as potential multitarget compounds for AD treatment.

Hybrid Compounds	Main Pharmacological Actions					References
**Donepezil-based hybrids**	Selectively and reversibly inhibits the human AChE and BChE.	Strong inhibition of aggregation of the Aβ peptide.	Protect neurons from the damage due to mitochondrial free radicals and have antioxidant activity.	Metal chelating action.	Increased potential to infiltrate BBB and reduced neurotoxicity.	[91,92,93]
**Rivastigmine-based hybrids**	Dual inhibition of AChE and BChE; specific monoamine oxidase-B (MAO-B) inhibitor.	Inhibit Aβ 1-42 aggregation, induced by Cu^2+^.	Anti-oxidative activity with reduced hepatotoxicity	Bio- metal chelating properties.	Provide neuroprotection. Favorable for BBB infiltration in vitro.	[94,95,96]
**Galantamine-based hybrids** (tertiary alkaloid of natural origin)	Strongly inhibit AChE activity in the synapse.	Decrease Aβ plaques.	Antioxidant and anti-inflammatory action.	Metal chelating action.	Cross the BBB by passive diffusion, and appear free of neurotoxicity.	[97,98,99]
**Carbamate-conjugated hybrids** (It has superior chemical stability due to the carbamic group, and ability to increase the permeability of biological membranes.)	Compound **6** has highly selective BChE inhibitory activity. Compound **7** demonstrated the highest AChEI activity by reversible noncompetitive partial inhibition.	Anti-amyloidogenic effect. Compound **8** strongly inhibits Aβ aggregation.	Have antioxidant-mediated neuroprotective activities.	Ability to chelate specific metals.	Better BBB permeability.	[100,101]
**Physostigmine-conjugated hybrids**	Tolserine and phenserine derived from physostigmine (PSM), are some non-competitive, selective and long-acting AChEI chemicals.	Reduce amyloid precursor protein (APP).	Tolserine is 200 times more selective for human AChE than for human BChE.	Phenserine is a selective AChEI with reduced side effects if compared to traditional AChEIs.	Phenserine has ability to inhibit Aβ-aggregation and was used for the treatment of cognitive impairment induced by traumatic brain injury in experimental studies.	[84,102,103]
**Rhein-Huprine-conjugated hybrids**	hAChE and hBChE are inhibited by synthesized rhein-huprine hybrids	Compound **16** is a therapeutic anti-Alzheimer candidate because it has multiple actions on hAChE, hBChE, BACE-1 and the accumulation of Aβ42.				[84]
**Novel Hybrid Therapeutic Compounds** **Targeting AD**	Biflavonoids significantly inhibited Aβ 1-42 fibrillization.	Amentoflavone (AMF) appears to have the greatest effect in suppressing fibrillization and complete disaggregation of Aβ 1-42 fibrils in AD.	Compound **17** would stimulate neurorejuvenation and arrest neurodegeneration in AD.	AMF can reduce extracellular Aβ by increasing its cellular uptake and clearance.	Berberine (BB), an isoquinoline alkaloid, might interfere with the pathogenic processes in AD by decreasing the levels of Aβ, blocking the activity of secretases in the APP pathway, reducing oxidative stress, astrocytosis and neuronal degradation.	[104,105,106,107,108,109]

**Table 2:** Characteristics of current FDA-approved drugs for Alzheimer’s therapy.

Generic Name and Year of Approval	Trade or Brand Names	Route of Administration and Doses	Clinical Benefits	Side Effects				References
				Gastrointestinal	Neuropsychiatric	Allergic Reactions	Other Effects	
**Mechanism of Action: Acetylcholinesterase Inhibitors (AChEIs)**								
**Donepezil** **1996**, FDA. **2022**, FDA: first transdermal system (Adlarity^®^).	**Aricept, Aricept ODT, Adlarity**, others	Orally standard dose (5–10 mg); high dose (23 mg). **Adlarity** as one transdermal patch applied to the skin once weekly.	A small benefit in mental function and ability to function.	Nausea; vomiting; diarrhea; loss of appetite; signs of stomach bleeding, etc.	Insomnia, or feeling tired; depression; hallucinations; convulsions; painful or difficult urination, etc.	Hives; difficulty breathing; swelling of face, lips, tongue, or throat, etc.	Increased creatine phosphokinase; dehydration; hyperlipemia; diabetes mellitus, goiter, liver dysfunction, etc.	[75] [92] [117,118,119,120,149] [151,152,153,154] [161,162,163,164,165]
**Rivastigmine** **2000,** FDA.	**Exelon** **^®^,** **Prometax, others**	Initial dose: 1.5 mg orally twice a day; can be increased to 3 mg twice a day; Transdermal patch: initial dose: 4.6 mg/24 h patch applied to the skin once daily; maximum dose: 13.3 mg/24 h.	Effective symptomatically in all types of dementia; allowing patients to be independent and “be themselves” for a long time.	Ulcer or stomach bleeding; vomiting; diarrhea; nausea etc.	Convulsions; tremors, jerky muscle movements in the eyes, tongue, jaw or neck.	Urticaria; difficult breathing; swelling of the face, lips, tongue or throat; severe skin redness, itching or irritation; asthma, etc.	Liver or kidney dysfunctions; dehydration symptoms. Heart and lung problems, etc.	[77] [94,95,96] [166,167,168,169] [170] [171,172,173,174] [175] [176]
**Galantamine** **2001**, FDA; **2003**, FDA: extended-release capsules.	**Razadyne Razadyne ER,** **Reminyl^®^** others	8–24 mg per day divided into 2 doses.	Improves cognition, function and activities of daily living.Delays the development of behavioral disturbances and psychiatric symptoms.	Nausea; vomiting; diarrhea; loss of appetite; upper abdominal pain, etc.	Tiredness; itching; headache; dizziness; feeling very thirsty or hot, etc.	Skin rash; hot and dry skin; progressive red or purple rash that causes blisters and peeling, etc.	Dark urine; clay-colored stools; jaundice with yellowing of the skin or eyes; blood in urine; bloody or tarry stools, etc.	[97,98,99] [121,122,123] [167] [177,178,179] [180,181]
**Mechanism of Action: NMDA Receptor Antagonist**								
**Memantine** **2002**, EMA. **2003**, FDA.	**Namenda** **Namenda XR, Axura, Ebixa,** others	Standard doses: 10–20 mg/daily (divided into 2 doses).	Moderately improves cognition, mood, behavior and ability to perform daily activities.	Vomiting; abdominal pain; diarrhea; loss of appetite; constipation; pancreatitis, etc.	Dizziness; headache; confusion; fatigue; pain; pain in the joints; lower back or muscle pain or stiffness; hallucinations; confusion; aggressive behavior; suicidal ideation, etc.	Swelling of the tongue, lips, or face; shortness of breath; skin rash; urticaria, etc.	Cardiac disorders—congestive heart failure. Hepatobiliary disorders—hepatitis. Renal and urinary disorders. Musculoskeletaldisorders, etc.	[80] [114,115,116,117,118,119,120] [182] [183] [184]
**Mechanism of Action: AChEI and NMDA Receptor Antagonist. Mixt Products.**								
**Memantine/Donepezil (Rx)** **2014,** FDA.	**Namzaric^®^**	Standard dose: 28 mg memantine/10 mg donepezil; once daily in the evening. 14 mg/10 mg for patients with severe renal impairment.	Namzaric may help improve cognition and global function in patients with moderate and severe forms of AD.	Vomiting; constipation; diarrhea; loss of appetite; abdominal pain; signs of stomach bleeding; severe heartburn or abdominal pain, bloody or tarry stools, etc.	Headache; dizziness; somnolence; anxiety; aggression; depression, etc.	Urticaria; difficult breathing; swelling of the face, lips, tongue, or throat, etc.	Cardiovascular disorders: slow heartbeats or chest pain; hypertension or hypotension; New or worsening breathing problems. Urinary incontinence. Bruises; coughing up blood, etc.	[118] [119] [120] [185]
**Mechanism of Action: Anti-amyloid Monoclonal Antibodies for Alzheimer′s Disease**								
**Aducanumab** **2021,** FDA. EMA **rejected** it in **2021**.	**Aduhelm, Aducanumab-avwa,** **BIIB037, BIIB-037.**	Administered standard as IV infusion 10 mg/kg every 4 weeks and at least 21 days apart.	It binds to Aβ oligomers and promotes their clearance, being able to reduce Aβ accumulation and slow the progression of cognitive impairment. A modest clinical benefit in AD, but significant adverse events (ARIA) and high cost. It has sparked benefit-risk controversies.	Nausea, diarrhea, etc.	Headache; vertigo; dizziness; altered mental status; confusion; incoherent talk; disorientation; seizures, etc.	Urticaria; difficulty breathing; swelling of the face, lips, tongue, or throat, etc.	Visual disturbances; ARIA-E: cerebral edema including greater sulcal effusion in carriers of apolipoprotein E4. ARIA-H: superficial siderosis; microhemorrhages, etc.	[121,122,123,124,125,126,127,128,129,130,131,132,133,134,135,136,137] [156] [186] [187]
**Lecanemab (Rx)** Accelerated approval on **6 January 2023** by the FDA.	**Leqembi, Lecanemab-irmb, BAN2401, mAb158**	**Intravenous infusion;** 10 mg/kg IV once every 2 weeks.	Lecanemab led to a rapid and pronounced decrease in amyloid plaques as well as a delay in clinical decline.	Nausea, vomiting, diarrhea, etc.	Headache; postural instability; confusion; incoherent talk; disorientation, etc.	Infusion-related reactions. Fever. Flu-like symptoms (chills, generalized aches, feeling shaky, and joint pain).	Atrial fibrillation; Hypotension or hypertension, oxygen desaturation, cough. Lymphopenia. ARIA-E (edema/effusion, etc.). ARIA-H (combined cerebral microhemorrhages, cerebral macrohemorrhages and superficial siderosis).	[139] [142] [145] [159] [160] [188] [170]

**Table 3:** Brain photobiomodulation studies in human subjects.

Reference	Type of Study and Modality of Work	Protocol of the Study	Study Results	Conclusions
[291]	Placebo controlled study for 30 (13 female; 17 male) students, mean age 20.4 yrs. 15 participants received active tPBM, and the other half, *placebo*. tPBM session lasted 8 min, administered in 8 one-minute treatments alternating between two locations on the forehead, each location was 4 cm in diameter. To quantify the effects of tPBM, the neuropsychological Wisconsin Card Sorting Test (WCST) was used, which is the gold-standard of executive function, including attention and memory.	A 1064 nm laser, 250 mW/cm^2^ (3400 mW/13.6 cm^2^ = 250 mW/cm^2^) was used for 4 min (3.4 W × 240 s = 816 J/location), which corresponded to an energy density of 60 J/cm^2^ (0.25 W/cm^2^ × 240 s = 60 J/cm^2^).	Study demonstrated that tPBM can improve cognitive function in healthy young adults in only 8 min.	tPBM with 1064 nm has proven experimentally that it can photostimulate CCO, the enzyme that catalyses oxygen consumption for energy production metabolism. It improved cognitive functions and would have an interesting potential for therapy or prevention of deficits from neuropsychological disorders or due to the aging process.
[292]	Case Series Report 5 old patients with “mild to moderate-severe” dementia and AD received tPBM and intranasal PBM with 810 nm, 10 Hz pulsed LEDs (41 transcranial and 23 intranasal diodes), 25 min/session at home, for 12 weeks.	Patients kept a “Daily Home Treatment Journal”, and at the clinic; changes in memory, cognition, general health conditions and any adverse effects were noted.	Mini-Mental State Exam (MMSE) and Alzheimer’s Disease Assessment Scale (ADAS-cog) scores improved significantly after 12 weeks of tPBM and intranasal PBM.	tPBM and intranasal PBM applied at home demonstrated the potential of this therapy in a small group of patients with dementia and AD.
[293]	Double-blind, placebo-controlled trial on 11 subjects with dementia (age 40–85 yrs.) treated in 28 daily sessions with tPBM with an IR device (1060–1080 nm) that had 1100 LEDs set in 15 arrays of 70 LEDs/matrix, pulsed at 10 Hz with a duty cycle of 50%, for a time of 6 min/day.	Patients were evaluated by a mini mental state examination (MMSE), quantitative EEG (QEEG) and ADAS-cog before and 3 days after the completion of treatment.	The results show a slight improvement in executive functioning; clock drawing, immediate recall, praxis memory, visual attention and task switching, as well as improved EEG amplitude and connectivity measures.	As a small pilot clinical trial using tNIR to increase mitochondrial ATP and induce neuronal plasticity, it did not reach statistical significance due to the short duration of therapy and small number of participants.
[294]	Randomized controlled trial on 21 subjects divided into two groups: 10 received tPBM twice a week in the frontal region, bilaterally via near-infrared (NIR) light-emitting diodes (LEDs) for 8 weeks), and 11 received sham tPBM.	28 LEDs [823 nm, CW; 28.7 × 2 cm^2^; 36.2 mW/cm^2^; up to 65.2 J/cm^2^; 20–30 min/session. Safety and efficacy were assessed using the modified Hamilton Depression Rating Scale (HAM-D_17_).	The study provided preliminary evidence for moderate to high antidepressant efficacy, as tested by the HAM-D_17_ total score, compared to the control group; tPBM was well tolerated, no serious adverse events.	tPBM with NIR light had a moderate to high antidepressant effect on the HAM-D_17_ scale.
[295]	30 older adults (≥60 years) without dementia were randomly assigned to two groups. tPBM device contained 9 R diodes of 633 nm wavelength and 52 NIR diodes of 870 nm, incorporated in 3 separate LED cluster heads (633 nm and 870 nm), with a total surface of 22.48 cm^2^; total power of 999 mW; power density of 44.4 mW/cm^2^ and CW emission. The tPBM group received the dose of 20 J/cm^2^, in 7.5 min/session, with a total energy dose of 1349 J, applied to both sides of the frontal region and the posterior midline.	The participants performed cognitive performance tests of frontal function (modified Eriksen flanker test and category fluency tests) before and after real or sham tPBM. The investigated parameters included: (1) CDRS, which estimates the level of global cognitive functioning. (2) CGDS which measures the level of depressive symptoms. (3) Beck Anxiety Inventory (BAI), which measures the level of anxiety symptoms; and (4) the Hong Kong List Learning Test (HKLLT).	tPBM significantly improved the action selection, inhibition ability, and mental flexibility after procedures vs. before tPBM, compared to placebo group.	tPBM could be used as a potential neuroprotective agent for preserving or repairing cognitive function in older adults, in a safe and cost-effective manner.
[296]	15 subjects (mean age 30–14 years; 67% women) suffering from generalized anxiety disorder (GAD) participated in an open-label 8-week study. Each participant self-administered t-PBM daily, for 20 min (CW; 830 nm; mean irradiance 30 mW/cm^2^; mean fluence 36 J/cm^2^; total energy delivered per session 2.9 kJ total output power; 2.4 W) on the forehead (total area 80 cm^2^) with an LED-cluster headband.	The monitored parameters included: the structured interview guide for The Hamilton Anxiety Scale (SIGH-A), Clinical Global Impressions-Severity (CGI-S) and—Improvement (CGI-I) subscales and the Pittsburgh Sleep Quality Index (PSQI).	tPBM had a significant effect in reducing the level of anxiety, with relatively few, mostly mild, and transient side effects.	tPBM could be an alternative therapy for patients with anxiety unresponsive to drugs or psychotherapy.
[297]	34 healthy adults (16 males, 18 females; average age: 31) were included in a double-blind randomized controlled study: 18 participants (9 male, 9 female) received transcranial infrared laser stimulation (TILS) and completed all tasks, performing the cognitive tasks before and after TILS, with concomitant fNIRS recordings, to reflect the hemodynamic effects of TILS on cognitive performance. 16 participants (7 male, 9 female) were matched blind as sham controls (TILS with light off).	Collimated laser diode: 1064 nm; CW mode; average radiant power: 3400 mW; irradiance 250 mW/cm^2^; beam spot size at forehead target 13.6 cm^2^; exposure duration: 480 s; radiant exposure: 120 J/cm^2^; radiant energy: 1632 J; number of points irradiated: one, non-contact; one session in 8 min. Performance on the psychomotor vigilance task (PVT) and the delayed match-to-sample task (DMS) were measured pre- and post-TILS. Functional near-infrared spectroscopy (fNIRS) was used to measure hemodynamics: concentration changes in oxygenated and deoxygenated hemoglobin, total hemoglobin, and differential effects.	fNIRS showed highly significant effects on prefrontal oxygenation during cognitive enhancement post-TILS.	Authors considered their study to be the first demonstration that cognitive enhancement by TILS is associated with cerebrovascular oxygenation of the prefrontal cortex.
[298]	22 elderly adults with mild cognitive impairment (MCI) were recruited through an online advertisement and divided into two groups. Inclusion criteria were no known history of head injury or epilepsy, psychological and/or neuropsychological disorders, or memory and/or other cognitive problem(s). They received tPBM in a single real or sham session. tPBM was administered to the forehead of each patient in the experimental and control group, respectively. For tPBM, a device with 16 probes, 9 LEDs with a wavelength of 810 nm, CW, irradiation power of 20 mW/cm^2^ was used, which was applied for 350 s, with a fluence of 7 J/cm^2^.	All subjects performed a visual memory test before and after tPBM measured with functional near-infrared spectroscopy (fNIRS).	tPBM improved the visual memory performance and decreased hemodynamic response during the tasks. tPBM may reduce the cognitive efforts needed to complete tasks that require high memory loads, and thus improved the cognitive performance.	tPBM can improve the cognitive performance of persons with MCI.
[299]	33 young healthy adults (16 males), with mean age of 25.24 years (SD = 8.86 years) were recruited and randomly assigned to control and experimental groups. A single PNM stimulation was applied to the forehead in the experimental group, while a sham PNM for the control group. PNM was performed with a helmet device on the participant’s forehead containing 5 LED clusters each with a spot area of 1 cm^2^, the wavelength of 810 nm, a power of 20 mW/cm^2^, an energy density of 7 J/cm^2^ in 350 s.	Before and after the stimulation, all participants performed an n-back task with 0-and 3-back conditions to assess their working memory function, and the hemodynamic responses during the tasks were measured by fNIRS. The investigated parameters included: verbal working memory ability (HKLLT); visual working memory (Rey–O); BAI; Changes in oxy-Hb and deoxy-Hb were recorded using a 16-channel fNIRS recording arranged in an array on each participant’s forehead.	Visual and verbal memory skills assessed by Rey-O were significantly correlated with oxy-Hb changes. Subjects receiving PNM had a significant improvement in visual memory performance and a reduced hemodynamic response measured by fNIRS during the visual memory task.	PNM may reduce the cognitive efforts needed to complete tasks with high memory loads. If an individual exerts less effort in performing a cognitive task after receiving a single PNM session, the level of oxy-Hb during that cognitive task will be reduced accordingly.
[300]	35 healthy participants over the age of 45 were recruited over two years, using age-matched participants (active group mean of age 57 ± 10 years; placebo group mean age of 57 ± 8 years). 27 participants completed the study. tPBM was performed at home for 6 min, 2 times a day. The helmet device was composed of 14 air cooled LED panel arrays with a wavelength of 1068 nm and a total average optical power output of 3.8 watts.	Computerized assessment of cognitive and motor activities was performed with the FDA-approved Automated Neuropsychological Assessment Metrics (ANAM) tool.	The results demonstrated a significant improvement in motor function, memory performance and processing speed compared to the placebo group. No adverse effects were reported.	tPBM may be the new method for improving memory in middle-aged people.
[301]	A single-blind, sham-controlled pilot study investigated the effect of continuous (c-tPBM), pulsed (p-tPBM), and sham (s-tPBM) transcranial photobiomodulation on EEG oscillations and CBF using diffuse correlation spectroscopy (DCS) for a sample of ten healthy subjects (6 F/4 M; mean age 28.6 ± 12.9 years). A NIR laser (830 nm; 54.8 mW/cm2; 65.8 J/cm^2^; 2.3 kJ) was used for c-tPBM and another for p-tPBM (830 nm; 10 Hz; 54.8 mW/cm^2^; 33%; 21.7 J/cm^2^); 0.8 kJ) were applied concurrently to the frontal areas by four LED clusters.	Simultaneous recordings of EEG and DCS were performed weekly before, during and after each tPBM session, as subjects rested (with no cognitive task). EEG was also recorded while participants performed a working memory (2-back) task: once at baseline, before the c-tPBM session, and after each tPBM session.	Use of c-tPBM significantly boosted gamma and beta EEG spectral powers in eyes-open recordings, and gamma power in eyes-closed recordings, with a widespread increase over frontal-central scalp regions. No significant effects of tPBM on CBF were found compared to sham.	The study results support the dose-dependent neuromodulatory effect of tPBM with NIR.
[302]	60 subjects (aged 50 to 85 years) diagnosed with mild to moderate AD/Alzheimer’s disease-related dementias (ADRD) and their primary caregivers were enrolled in a randomized, double-blind, controlled study, at a 2:1 ratio to the active arm or the control arm (sham). Subjects received either an active wearable PBM unit or a sham wearable unit to be used at home twice a day for six minutes, for eight consecutive weeks. tPBM device contained 12 LED modules covering the skull and two retractable modules to provide intraocular stimulation. Each skull module had 70 LEDs and each eye module had 14 LEDs. The active PBM device emitted NIR light with a wavelength of 1060–1080 nm, 15,000 mW, 23.1 mW/cm^2^ irradiance and for a treatment area of ~650 cm^2^.	2 neuropsychological assessments were conducted 8 weeks apart. Evaluation of cognitive function was carried out through: MMSE—which evaluates concentration, orientation, language, attention, memory and visual-spatial function; the clock drawing test (CDT); clock copy test (CPT); Logical Memory Test—Immediate Recall (LMT-I); Logical Memory Test—Delayed Recall (LMT-II); The route making test A; Trail Making Test B; Boston Naming Test (BNT), WAIS-R Digit Symbol Substitution Test.	The 39 randomized subjects in the active arm who remained in the study reported that their energy and mood increased, anxiety decreased, and they had a greater ability to participate physically and mentally in daily activities.	At the end of the study (8 weeks), all subjects remaining in the active arm, both men and women, showed better cognitive performance than those in the control arm.
[303]	60 subjects (of whom 57 completed the study) with mild to moderate dementia were included in a placebo controlled, randomized, double-blinded trial for active treatment with low power NIR light radiation for 6 min twice a day for 8 consecutive weeks. Active and simulated headsets had 12 cranial modules with 70 LEDs/module and 2 foldable eye modules with 14 LEDs/module. The devices issued NIR light at the wavelength of 1060–1080 nm and power of 15,000 mW, the power density of 23.1 mW/cm^2^, ~650 cm^2^ per treatment area.	Neuropsychological monitoring was performed by the Alzheimer’s Disease Neuroimaging Initiative (ADNI) test, MMSE, ADAS-cog, CDT, auditory-immediate verbal learning test (A.V.L.T.-1 and A.V.L.T.-2), digit span forward and backward (DSF and DSB), trail making tests A and B and WAIS-R test.	The results showed that this treatment improved cognitive functions, logical memory, auditory verbal learning, mood, sleep duration and daily routine energy in patients with dementia.	tPBM has confirmed the beneficial role in improving the quality of life and self-independence of patients with dementia, reducing the burden on family caregivers. tPBM had no local or systemic adverse effects. Much more studies are needed for the routine application of tPBM.
[304]	In a placebo-controlled clinical trial, 60 elderly patients with anemia and mild cognitive dysfunction received intranasal and transcutaneous radial artery PBM (tc-RA-PBM) with a 650-nm clock laser, acupuncture, and moderate-intensity aerobic exercise for 12 weeks.	Monitored parameters: Hb level, cognition by the Montreal Cognitive Assessment Scale (MoCa—B basic), Quality of Life for Alzheimer’s Disease scale and Berg Balance scale scores together, body mass index (BMI) and waist-to-hip ratio (WHR).	PBM showed more significant results compared to the control group in all the measured outcomes.	Intranasal PBM combined with wrist acupuncture and moderate-intensity aerobic exercise might be more effective in improving cognitive function and quality of life in AD patients.
[305]	68 healthy subjects aged 18 to 85 years were included in a randomized, sham-controlled trial. Broadband near-infrared spectroscopy was used for the noninvasive quantification of bilateral cortical changes in oxidized cytochrome-c-oxidase and hemoglobin oxygenation before, during and after 1064-nm PBM (NIR-laser, area: 13.6 cm^2^, power density: 250 mW/cm^2^) or sham stimulation of the right anterior prefrontal cortex (Brodmann Area 10).	Montreal Cognitive Assessment (MoCA) measured global cognitive functioning. A non-invasive broadband NIRS (bbNIRS) system was customized to measure the concentration of oxidized CCO (Δ[CCO]), oxygenated hemoglobin (Δ[HbO]) and deoxygenated hemoglobin (Δ[Hb]) in the prefrontal cortex (PFC).	Results showed a significant increase in Δ[CCO] during laser stimulation, followed by a significant post-stimulation increase in Δ[HbO and a decrease in Δ[Hb]. No adverse effects of tPBM were found.	The findings support the use of tPBM for cerebral oxygenation and attenuating the age-related decline of mitochondrial respiration.
[306]	54 subjects with major depressive disorder (MDD) were included in a 2-site, double-blind, sham-controlled study, conducted for adjunct tPBM in NIR (830 nm; CW; 35.8 cm^2^ treatment area; 54.8 mW/cm^2^ irradiance; 65.8 J/cm^2^ fluence, 20 min/session; ~2 W total power; 2.3 kJ total energy per session), delivered to the prefrontal cortex, bilaterally, twice a week for 6 weeks. 18 non-responders to sham in phase 1 (6 weeks) were re-randomized in phase 2.	Patients were assessed using the Hamilton Depression Rating Scale [HDRS-17] and the Quick Inventory of Depressive Symptomatology-Clinician Rating [QIDS-C] score.	In the primary outcome, results showed decreases in depression severity on the HDRS-17 scale and QIDS-C scores.	This study suggests the efficacy of tPBM for patients with MDD; but cannot specify the optimal effective dose.
[307]	A case study for an elderly person with a Self-Administered Gerocognitive Exam (SAGE) score indicating memory and thinking disorder. tPBM was administered by a 4-clusters of 3 IR LEDs (810 nm) and 4 levels of power. Stimulations were performed over a period of 35 days at a frequency of 10 Hz. In the first week, 3 sessions were performed at power level 2, then five sessions per week at power level 3, producing a power density of 4.2, respectively 12.7 mW/cm^2^ for each of the 3 clusters of LEDs.	EEG recorded for 10 min before tPBM in two conditions with eyes open and eyes closed for 5 min each; EEG during PBM and 10 min after stimulation.	PBM could have positive effects on brain activity in the theta and alpha bands for older people with memory and thinking disorders.	PBM has positive effects on brain activity, measured as improvement in power spectrum and connectivity in the theta and alpha bands for older people with memory and thinking disorders.
[308]	4 Case Studies with dementia (two with mild to moderate dementia and two with more advanced symptoms), received tPBM at home. A high-powered super-pulsed laser with 5–905 nm (maximum 200 mW) and 4–660 nm (75 mW -max. 100 mW) diodes was used. Patients were treated three times over a five-day period. The energy delivered per site was 144 J, in 6 areas: four areas on the pre-frontal cortex and two areas on the mid-brain. The total energy delivered over all six sites was 864 J.	Response to tPBM was assessed by MMSE, except for cases with advanced dementia.	Results suggest that tPBM with a high-powered super-pulsed laser applied for three or four eight-minute treatments over a 5–7-day period when using super-pulsing technology could significantly improve cases of moderate and advanced dementia.	Super-pulsed laser devices with higher power could provide improvements in a shorter period of time in AD and dementia. This non-invasive, non-pharmaceutical, and safe treatment approach should be more widely adopted.
[309]	In a tPBM study (with a laser of 1064-nm or sham), the EEG data sets were recorded and analyzed from a total of 44 healthy human subjects on a 64-channel EEG before, during, and after 8-min, on the right-forehead tPBM application, and the data were processed with a novel methodology by combining group singular value decomposition (gSVD) with the exact low-resolution brain electromagnetic tomography (eLORETA), implemented and performed on the 64-channel noise-free EEG time series. The gSVD + eLORETA algorithm produced gSVD-derived principal components (PCs) projected in the 2D sensor and 3D source domain/space. Finally, baseline-normalized power changes of each EEG brain network in each EEG frequency band (delta, theta, alpha, beta and gamma) were quantified during the first 4-min, second 4-min, and post tPBM/sham periods, followed by comparisons of frequency-specific power changes between tPBM and sham conditions.	tPBM was conducted with a 1064-nm laser (illumination area of 13.6 cm^2^, power of 3.5 W, laser aperture diameter of ∼4.16 cm). Active optical energy (or dose) was 3.5 W × 480 s = 1680 J and energy density (or fluence) delivered to the forehead was 1680 J/(13.6 cm^2^) = 123.5 J/cm^2^, respectively. Active power density (irradiance) was 3.5 W/(13.6 cm^2^) = 257.4 mW/cm^2^. Power used for sham was set to be 0.1 W.	Results highlighted that 1064-nm tPBM applied on right-forehead, could neuromodulate the alpha and gamma powers on several of the gSVD-derived EEG brain networks, i.e., the well-defined (MRI-derived): default-mode network, frontalparietal network, and executive control network.	This study clearly proved mechanistic associations or causal effects of tPBM and modulated brain networks versus improved cognition outcomes.
[310]	Effect of PBM on regression of dementia and cognitive impairment in various AD stages was studied on 97 patients with previously diagnosed AD, aged 34–80 (mean age 67.5), divided into two groups: Test Group (G1)—48 patients, treated with Transcatheter Intracerebral PBM (PBMT), and Control Group (G2)—49 patients who underwent conservative treatment with Memantine and Rivastigmine. PBMT was administered with a 632.8 nm laser device; laser output power of 25–45 mW; fiber output power of 24–44 mW; stream duration of 1200–2400 s; diameter of the laser beam in the vessel of 1–2 mm, average dose of 29–106 J.	Examinations of patients were carried out as follows: the clinical severity of dementia was assessed using CDR, the cognitive impairment using MMSE, cerebral blood flow and cerebral microcirculation using SG in static and dynamic modes; cerebral perfusion blood filling with REG, and intracerebral vascular and capillary bed was evaluated by MUGA. Cerebral structural and morphological changes were assessed using CT and MRI performed upon the patients’ admission, then, at an interval of 6–12 months. Examinations were performed using the digital image processing program ATAA and digital morphometric scale TDR.	As a result of PBMT, involutive changes were reduced, the normal structure of the cerebral tissue was restored, and the volumes of the temporal and frontoparietal lobes increased. Decreased dementia severity and restoration of cognitive functions in AD patients were found in G1. Conservative treatment in the control group did not stimulate regenerative processes and did not improve microcirculation.	PBMT is an effective, physiologically based method of stimulating cerebral angiogenesis and neurogenesis. Tissue regeneration in G1 led to an increase in temporal and frontoparietal volume, a stable decrease in the level of dementia, restoring cognitive functions and improving patients’ quality of life. This clinical effect was maintained for many years. Treatment with Memantine and Rivastigmine was not effective.
[311]	Case studies. Three older adults with non-amnestic MCI received 18 sessions of tPBM stimulation over 9 weeks (i.e., twice per week). A device with 9 individual LED nodes of 1 cm^2^ size, were placed cranially in the frontal area. Each LED emits 810 nm light, at an irradiance of 20 mW/cm^2^, for 350 s, with a fluence of 7 J/cm^2^, total surface area of 9 cm^2^, energy delivered per session was 189 J and the total energy delivered for the 18 sessions was 3402 J.	Patients were assessed using CDRS, CDR, FAQ, HKLLT, Rey-O, CGDS-SF, GAS-10, CFT.	tPBM applied to the frontal lobe area during 18 sessions improved cognitive functions, depressive and anxiety symptoms in three elderly adults with MCI.	This study provided preliminary support for tPBM as a non-invasive intervention to improve cognitive functions and mental health in elderly people with MCI. Further investigation through larger randomized placebo-controlled studies is needed to confirm tPBM potential.

## Data Availability

The references used to support the findings of this study are available from the first (L.M.A.) and the corresponding (G.L.) authors upon request.

## Abbreviations

Acetylcholine	(ACh)
Acetylcholinesterase	(AchE)
Acetylcholinesterase inhibitor	(AChEI)
AD assessment scale-cognitive subscale	(ADAS—cog)
Adenosine diphosphate	(ADP)
Adenosine triphosphate	(ATP)
Advanced Tomo Area Analysis	(ATAA)
Alpha (Greek letter, lowercase alpha)	(α)
Alzheimer’s Disease	(AD)
Alzheimer’s Disease-related dementias	(ADRD)
Alzheimer’s Disease Neuroimaging Initiative	(ADNI)
Amyloid beta	(Aβ)
Amyloid precursor protein	(APP)
Amyloid-related imaging abnormalities	(ARIAs)
Amyloid-related imaging abnormalities of effusion	(ARIA-E)
Amyloid-related imaging abnormalities of hemorrhagic	(ARIA-H)
Antibody	(Ab)
3D6 antibody fragments	(3D6-Fab)
Antigen presenting cell	(APC)
Auditory-immediate verbal learning test 1	(A.V.L.T.-1)
Auditory-immediate verbal learning test 2	(A.V.L.T.-2)
Automated Neuropsychological Assessment Metrics	(ANAM)
Aβ oligomers	(AβOs)
Beck Anxiety Inventory	(BAI)
Beta tubulin III	(Tuj-1)
Blood–brain barrier	(BBB)
Body mass index	(BMI)
Boston Naming Test	(BNT)
Brain extracellular space	(ECS)
Brain interstitial fluid	(ISF)
Brain-derived neurotrophic factor	(BDNF)
Broadband near-infrared spectroscopy	(bbNIRS)
Butyrylcholinesterase	(BChE)
Carboxy-terminal fragment	(CTF)
Category Fluency Test	(CFT)
CD4+ T lymphocytes	(CD4+)
CD8+ T lymphocytes	(CD8+)
Central nervous system	(CNS)
Cerebral blood flow	(CBF)
Cerebrospinal fluid	(CSF)
Chinese version of the Dementia Rating Scale	(CDRS)
Chinese version of the Geriatric Depression Scale	(CGDS)
Chinese version of the Geriatric Depression Scale—Short Form	(CGDS-SF)
Cholinesterase	(ChE)
Cholinesterase inhibitors	(ChEIs)
Clinical Dementia Rating Scale	(CDR)
Clinical Global Impressions Improvement subscale	(CGI- I)
Clinical Global Impressions-Severity	(CGI-S)
Clock copy test	(CPT)
Clock drawing test	(CDT)
Computed tomography	(CT)
Concentration changes of oxidized CCO	(Δ[CCO])
Concentration changes of deoxygenated hemoglobin	(Δ[Hb])
Concentration changes of oxygenated hemoglobin	(Δ[HbO])
Continuous wave	(CW)
Continuous tPBM	(c-tPBM)
Cyclic adenosine monophosphate	(cAMP)
Cytochrome c oxidase	(CCO)
Decrease	(↓)
Delayed match-to-sample task	(DMS)
Deoxyribonucleic acid	(DNA)
Diffuse correlation spectroscopy	(DCS)
Diffusion rate in ECS-mapping or Diffusion rate in brain extracellular space	(D_ECS_-mapping)
Digit span backward	(DSB)
Digit span forward	(DSF)
Direction of paracellular transport	↓↓
Donepezil	(DP)
Drug delivery	(DD)
Drug delivery systems	(DDSs)
Electroencephalogram	(EEG)
Electron transport chain	(ETC)
Erythrocyte	(E)
European Medicines Agency	(EMA)
Excitatory field potentials/field excitatory postsynaptic potentials	(fEPSPs)
Extended release	(ER)
Flavin mononucleotide	(FMN)
Flavin-adenosine-dinucleotides	(FADs)
U.S. Food and Drug Administration	(FDA)
Functional Activities Questionnaire	(FAQ)
Functional near-infrared spectroscopy	(fNIRS)
Galantamine	(GAL)
Galantamine nanoparticles	(G-NPs)
Generalized anxiety disorder	(GAD)
Geriatric Anxiety Scale-10-item Version	(GAS -10)
Geriatric Depression Scale	(GDS)
Gold nanoparticles	(AuNPs)
Granulocyte-macrophage colony stimulating factor	(GM-CSF)
Hamilton Anxiety Scale	(SIGH-A)
Hamilton Depression Rating Scale	(HDRS-17)
Hamilton Depression Rating Scale (modified)	(HAM-D_17_)
Hong Kong List Learning Test	HKLLT
Human acetylcholinesterase	(hAChE/huAChE)
Hydrogel-loaded NP systems	(NLH)
Hydrogen peroxide	(H_2_O_2_)
Increase	(↑)
Insulin-like growth factors-1	(IGF-1)
Interferon-γ	(IFN-γ)
Interleukin 1-beta	(IL-1β)
IL2 signal sequence	(IL2ss)
Interleukin-6	(IL-6)
Interleukin-10	(IL-10)
Janus kinase inhibitor	(JAK)
Light Amplification by Stimulated Emission of Radiation	(LASER)
Light emitting diode	(LED)
Logical Memory Test—Immediate Recall	(LMT-I)
Logical Memory Test—Delayed Recall	(LMT-II)
Long-term depression	(LTD)
Long-term potentiation	(LTP)
Lower generation PAMAM and lactoferrin conjugate	(PAMAM-Lf)
Low-level laser (or light) therapy	(LLLT)
CD4+ T lymphocytes	(CD4+)
CD8+ T lymphocytes	(CD8+)
Activated or healing macrophage of the M2 type	(M2)
Magnetic resonance angiography	(MRA)
Magnetic resonance imaging	(MRI)
Major depressive disorder	(MDD)
Memantine	(MEM)
Messenger ribonucleic acid	(m)RNA
Methoxy poly(ethylene glycol)-*co*-poly(ε-caprolactone)	(mPEG-PCL)
Microwave Amplification by Stimulated Emission of Radiation	(MASER)
Mild cognitive impairment	(MCI)
Mini mental state examination	(MMSE)
Mitochondria/mitochondrial	mt
Mitochondrial membrane potential	(MMP)
Modification of the Hamilton Depression Rating)	(HAM-D_17_)
Monoamine oxidase-B	(MAO-B)
Monoamine oxygenase	(MAO)
Monocyte	(Mo)
Montreal Cognitive Assessment Scale	(MoCa—B basic)
Morris Water Maze	(MWM)
Multi-gated angiography cerebral	(MUGA)
Multi-target-directed ligands	(MTDLs)
Multi-target-directed ligands	(MTDLs)
NAD(P)H quinone oxidoreductase 1	(NQO1)
Nanoemulsion	(NE)
Nanoparticle	(NP)
Nanostructured lipid carriers	(NLC)
Natural Killer cell	(NK cell)
Near-infrared	(NIR)
Neural Stem Cells	(NSCs)
Neurofibrillary structures/tangles	(NFTs)
Neuropsychiatric Inventory	(NPI)
Nitric oxide	(NO)
*N*-methyl-d-aspartate receptors	(NMDARs)
NPs-loaded hydrogel	(NLH)
Oligomers Tau	(TauOs)
Oxidized CCO	(oxCCO)
Oxygenated hemoglobin	(HbO)
Paired pulse facilitation	(PPF)
Parkinson’s disease	(PD)
Phosphorylated tau	(P-tau)
Photobiomodulation	(PBM)
Transcatheter Intracerebral PBM	(PBMT)
Photodynamic inactivation	(PDI)
Photodynamic therapy	(PDT)
Photoneuromodulation	(PNM)
Photosensitizer	(PS)
Photothermal therapy	(PTT)
Picosecond	(ps)
Pittsburgh Sleep Quality Index	(PSQI)
Poly(amidoamine)	(PAMAM)
Poly(d,l-lactide-*co*-glycolide) (50:50)-*b*-poly(ethylene glycol)	[PLGA-*b*-PEG]
Polymorphonuclear neutrophil	(PMN)
Positron emission tomography	(PET)
Postsynaptic density protein 95	(PSD-95)
Prefrontal cortex	(PFC)
Presenilin-1	(PSEN-1)
Presenilin-2	(PSEN-2)
Psychomotor vigilance task	(PVT)
Pulsed tPBM	(p-tPBM)
Quantitative EEG	(QEEG)
Quick Inventory of Depressive Symptomatology-Clinician Rating	(QIDS-C)
Reactive oxygen species	(ROS)
Red	(R)
Red-light treatment	(RLT)
Rey–Osterrieth complex figure test	(Rey–O)
Rheoencephalography	(REG)
Rivastigmine	(RSM)
Scanning electron microscopy	(SEM)
Scintigraphy	(SG)
Self-Administered Gerocognitive Exam	(SAGE)
Sham tPBM	(s-tPBM)
Signal transducer and activator of transcription 4	(STAT4)
Signal transducer and activator of transcription 5	(STAT5)
Silver nanoparticles	(AgNPs)
Single-chain variable fragment	(scFv)
Soluble amyloid precursor protein α	(sAPPα)
Soluble amyloid precursor protein β	(sAPPβ)
Synaptic plasticity	(SP)
Tacrine	(TAC)
Theta (Greek letter, lowercase theta)	(θ)
Tight junction	(TJ)
Tomography Dementia Rating	(TDR)
Total tau	(T-tau)
Transcranial infrared laser stimulation	(TILS)
Transcranial light therapy	(TLTC)
Transcranial near-infrared	(tNIR)
Transcranial PBM	(tPBM)
Transcutaneous radial artery PBM	(tc-RA-PBM)
Transforming growth factor-β1	(TGFβ1)
Tumor necrosis factor alpha	(TNF-α)
Ultraviolet A	(UVA)
Ultraviolet B	(UVB)
van der Waals	vdW
Waist–hip ratio	(WHR)
Wild-type	(WT)
Wisconsin Card Sorting Test	(WCST)
World Health Organization	(WHO)
